# Variabilities in global DNA methylation and β-sheet richness establish spectroscopic landscapes among subtypes of pancreatic cancer

**DOI:** 10.1007/s00259-023-06121-7

**Published:** 2023-02-09

**Authors:** Krzysztof Szymoński, Ewelina Lipiec, Kamila Sofińska, Katarzyna Skirlińska-Nosek, Michał Czaja, Sara Seweryn, Natalia Wilkosz, Giovanni Birarda, Federica Piccirilli, Lisa Vaccari, Łukasz Chmura, Joanna Szpor, Dariusz Adamek, Marek Szymoński

**Affiliations:** 1 Department of Pathomorphology, Jagiellonian University Medical College, Grzegorzecka 16, 31-531, Cracow, Poland; 2 Department of Pathomorphology, University Hospital, Cracow, Poland; 3 M. Smoluchowski Institute of Physics, Jagiellonian University, Cracow, Poland; 4 Elettra-Sincrotrone Trieste S.C.P. A, Trieste, Italy

**Keywords:** DNA methylation, Beta-sheet richness, Pancreatic cancer subtyping, Raman spectroscopy, Molecular imaging, Neural networks

## Abstract

**Purpose:**

Knowledge about pancreatic cancer (PC) biology has been growing rapidly in recent decades. Nevertheless, the survival of PC patients has not greatly improved. The development of a novel methodology suitable for deep investigation of the nature of PC tumors is of great importance. Molecular imaging techniques, such as Fourier transform infrared (FTIR) spectroscopy and Raman hyperspectral mapping (RHM) combined with advanced multivariate data analysis, were useful in studying the biochemical composition of PC tissue.

**Methods:**

Here, we evaluated the potential of molecular imaging in differentiating three groups of PC tumors, which originate from different precursor lesions. Specifically, we comprehensively investigated adenocarcinomas (ACs): conventional ductal AC, intraductal papillary mucinous carcinoma, and ampulla of Vater AC. FTIR microspectroscopy and RHM maps of 24 PC tissue slides were obtained, and comprehensive advanced statistical analyses, such as hierarchical clustering and nonnegative matrix factorization, were performed on a total of 211,355 Raman spectra. Additionally, we employed deep learning technology for the same task of PC subtyping to enable automation. The so-called convolutional neural network (CNN) was trained to recognize spectra specific to each PC group and then employed to generate CNN-prediction-based tissue maps. To identify the DNA methylation spectral markers, we used differently methylated, isolated DNA and compared the observed spectral differences with the results obtained from cellular nuclei regions of PC tissues.

**Results:**

The results showed significant differences among cancer tissues of the studied PC groups. The main findings are the varying content of β-sheet-rich proteins within the PC cells and alterations in the relative DNA methylation level. Our CNN model efficiently differentiated PC groups with 94% accuracy. The usage of CNN in the classification task did not require Raman spectral data preprocessing and eliminated the need for extensive knowledge of statistical methodologies.

**Conclusions:**

Molecular spectroscopy combined with CNN technology is a powerful tool for PC detection and subtyping. The molecular fingerprint of DNA methylation and β-sheet cytoplasmic proteins established by our results is different for the main PC groups and allowed the subtyping of pancreatic tumors, which can improve patient management and increase their survival. Our observations are of key importance in understanding the variability of PC and allow translation of the methodology into clinical practice by utilizing liquid biopsy testing.

**Supplementary Information:**

The online version contains supplementary material available at 10.1007/s00259-023-06121-7.

## Introduction

Some of the most aggressive and deadly malignant neoplasms arise within the head of the pancreas and can pose diagnostic problems even for experienced pathologists [1]. Pancreatic cancer (PC) is the fourth most lethal neoplasm in the USA [2] and seventh in the world [3]. A total of 49,830 deaths from PC were estimated in 2022 in the USA [2]. The 5-year survival rates are still below 10% [4]. One of the reasons for this persistent fact is the known heterogeneity in both molecular and morphological phenotypes, which is not properly reflected in current treatment options and causes the tumor to be largely chemoresistant. The lack of specific and sensitive early diagnostic methods leads to late-stage disease at the time of diagnosis [4]. New molecular detection and differentiation methods must be introduced to increase PC patient survival. For this purpose, it is crucial to use a systemic approach, with accurate and detailed histomorphological subtyping of samples.

Raman hyperspectral mapping (RHM) is a molecular imaging technique that involves Raman spectroscopy measurements of adjacent parts of the studied sample, resulting in a tissue map image. A first-ever Raman map of ampullary cancer was recently described in [5]. In the current study, we make significant progress in exploring the potential of RHM for pancreatic tumor characterization and show the RHM-based distinction among the three main types of pancreatic malignancies. Specifically, we investigated conventional pancreatic ductal adenocarcinoma (cPDAC), intraductal papillary mucinous carcinoma (IPMC), and ampulla of Vater adenocarcinoma (AVAC). These tumors are treated similarly in terms of clinical management, but recent reports suggest that they differ in prognostic factors occurrences, such as tumor differentiation level, perineural and venous invasion, and lymph node involvement [6], resulting in variations in PC patient survival.

There are two main pathways of carcinogenesis in the pancreas [4]. The first is called pancreatic intraepithelial neoplasia (PanIN), and the second develops based on a benign lesion called intraductal papillary mucinous neoplasm (IPMN—contrary to IPMC). PanIN is a dysplastic change of the pancreatic ductal epithelium, either in “normal” pancreatic tissue or in so-called acinar-to-ductal metaplasia regions [7]. It is considered the main precursor lesion for cPDAC [4]. On the other hand, IPMC arises in a neoplastic lesion that grows inside the pancreatic ducts, causing them to form a mucinous cystic tumor, called IPMN. The dysplastic lesions of the IPMN epithelium progress from low grade to high grade and eventually achieve invasive potential, transforming into cancer (IPMN evolves into IPMC). The initiation and progression pathways of these two precursor lesions differ in many aspects, although they overlap in others [8]. Another tumor involving the pancreatic head is ampullary cancer or ampulla of Vater cancer of the duodenum (AVAC), which originates via dysplastic changes in the epithelium of either the intestinal mucosa (intestinal type AVAC), the pancreatobiliary ductal mucosa (pancreatobiliary type AVAC), or both (mixed type AVAC) [8].

Currently, the distinction among cPDAC, IPMC, and AVAC is recognized by pathologists mainly by the location of the tumor epicenter (AVAC) and by the assessment of specific morphological features (cPDAC, IPMC) [4]. However, in many cases, they occur as a mixture of colorectal and pancreatic cancers [9]. Correct diagnosis is often difficult, especially when the AVAC infiltrates deep into the pancreatic tissue [1, 8] or when the so-called “large duct/cystic papillary” pattern of cPDAC occurs, which can hinder differentiation from IPMC [4]. The lack of specific and sensitive histopathological ancillary studies does not support making the diagnostic decision. Immunohistochemistry often gives ambiguous results [8]. Nevertheless, it is crucial to make the correct diagnosis, as it affects patients’ prognosis and care [6]. Notably, Reid et al., in a study on a large cohort of 232 AVAC and 476 cPDAC cases, showed significantly shorter survival of cPDAC patients compared to the worst type of AVAC, which is the pancreatobiliary type (15.6 vs. 41 months), regardless of the lymph node status and the tumor size [10]. Here, we present a new method of testing PC tissues that reveals the site of the cancer origin, thus helping to identify patients with varied prognoses but, most importantly, better adjusting the therapeutic management to the PC subtype.

Different methods of molecular vibrational spectroscopy (VS) were shown to be usable for characterizing the chemical structure of malignant tissues [11, 12]. Raman spectroscopy (RS) can become an efficient tool supporting the early diagnosis of pancreatic malignancy [5, 13]. Nevertheless, currently, the lack of screening methods leads to late-stage disease at diagnosis and consequently poor PC patient survival [14]. The interaction of the analyte with the light (electromagnetic radiation), which takes advantage of inelastic scattering, allows reading information about the composition of biologically significant molecules and functional groups, such as phosphates, proteins, carbohydrates, phospholipids, triglycerides, and nucleic acids. The results of such experiments allow conclusions to be drawn about differences in the metabolic pathways of various neoplasms [15]. One of the main advantages of molecular spectroscopic methodology is collecting information on samples in a label-free and noninvasive manner, which makes it a good candidate for an early, serum-based diagnostic tool for PC [14]. On the other hand, the data about samples obtained with RS methodology allows a deep investigation of their molecular composition. Unlike other methods, such as mass spectrometry proteomics, RS cannot identify specific proteins or discover new biomarkers. The key to success lies in identifying the “fingerprint” of samples. Although many factors can interfere with this “global picture,” such as inflammation or tumor necrosis, RHM allows the precise analysis of data within the nuclei or cytoplasm of cancerous cells, limiting the possibility of falsification [5]. Neural networks are exceptionally efficient in reading the smallest differences in that “fingerprint.”

Many publications have described the successful VS-based detection of cancer in organs such as the lung [16], bile duct [17], ovary [18], breast [19], or pancreas [20] by testing blood serum samples. None of them, however, compared spectral results in various cancers, and thus the true specificity of this methodology has not been assessed. Our study and obtained results show the utility of VS in differentiating various types of cancers. Although we characterized the spectral differences between PC groups in tissue samples, this knowledge can be directly translated into the interpretation of blood serum analysis by other FTIR or Raman-based methods.

To initiate the overview of insights into the PC biology that molecular spectroscopy might provide, we utilized Fourier transform infrared (FTIR) microspectroscopic imaging of PC samples. Knowledge of the spatial distribution of cancerous and noncancerous components of studied samples permitted the cognitive collection of RHM maps. Subsequently, we employed advanced statistical tools to extract spectral markers of PC and explore the spectral differences among them. Furthermore, as a proof of concept of utilizing VS-based methods in the development of early PC diagnostic and subtyping technology, which requires automation, we studied the potential of deep networking, specifically conventional neural networks (CNNs), to distinguish AVAC, IPMC, and cPDAC from raw spectra selected from RHM maps. This approach allowed automatic spectral classification without the need for human-dependent preprocessing or manual re-evaluation.

First, however, the investigations in this project addressed the biomolecular context of PC. We aimed to introduce the epigenetically complex reality of PC and its influence on protein modifications. The goal was to extend beyond “single-gene” genetics and transcriptomics with their (not unsubstantial) hope to decipher the still-elusive “language” of these most malignant neoplasms and to find common and specific differences in their observable biomolecular signatures. VS might be an excellent tool supporting such an approach.

## Methods

### Tissue slide preparation

Molecular imaging of 24 PC tissue slides from 17 patients was conducted. Specifically, 6 AVAC, 7 cPDAC, 9 IPMC, and 2 benign specimens were included. Patients with a diagnosis of PC who underwent pancreatoduodenectomy (Whipple or Traverso) or distal pancreatectomy were included in the study. Patients with a benign pancreatic neoplasm or neuroendocrine neoplasm were excluded. Tissue samples were selected from the Cracow University Hospital’s Pathomorphology Department’s archive, normally stored as conventional formalin-fixed paraffin-embedded (FFPE) blocks after the diagnostic process. Standard hematoxylin–eosin-stained glass slides (H&E) were used for initial sample selection, which was performed by two independent experienced pancreatic pathologists with the use of a routine light microscope (Olympus BX53 Microscope, RRID:SCR_022568). During the selection process, a detailed reevaluation of the tumor type was conducted. All initial diagnoses were confirmed. For each selected case, a single 2.5 μm thick tissue section was sliced with a Microm® HM355S Automatic Microtome and mounted onto a CaF_2_ window (Raman Grade Calcium Fluoride substrates—CRYSTRAN LTD, England). Subsequently, on unstained CaF_2_ slides, areas of interest including cancerous cells and the stroma compartment were marked by pathologists. Then, a complete paraffin removal procedure was conducted involving a 12-h xylene bath and graded ethanol rehydration. An overview of the patients included in the study is summarized in Supplementary Table S1.

### FTIR data acquisition

The initial FTIR measurements were executed on preprocessed pancreatic tissue slides (according to the procedure provided in the above “Tissue slide preparation” section). FTIR images were collected at the Chemical and Life Science branch of the infrared Beamline SISSI, Elettra Sincrotrone Trieste (Trieste, Italy), using a Hyperion 3000 Vis–IR microscope equipped with a liquid nitrogen-cooled bidimensional focal plane array (FPA) detector (64 × 64 pixels) coupled with a Vertex 70 v interferometer (Bruker Optics GmbH, Ettlingen, Germany). The IR data were acquired in transmission mode with a 15 × objective. Spectra were registered in the spectral range from 4000 to 900 cm^−1^ with a spectral resolution of 4 cm^−1^, averaging 256 scans. The FTIR images were acquired with a pixel size of 2.8 µm, and all the acquired maps covered the submillimeter areas of tissue sections (from 360 µm × 360 µm to 720 µm × 720 µm).

### Raman measurements

After recognizing the spatial distribution of PC in the studied samples, Raman measurements were executed using a Horiba LabRam spectrometer equipped with a green (532 nm) laser and electron-multiplying charge-coupled device (EM-CCD) camera cooled to − 70 °C. During the measurements, the tissue sections were immersed in a physiological saline solution, and a × 60 water immersion objective lens (Nikon) was used. Spectra were acquired in the fingerprint spectral region (1900–600 cm^−1^) with a spectral resolution of 2 cm^−1^. The process of RHM relies on multiple measurements of adjacent “pixels” of tissue and combining the resulting spectra into a single map image. In this study, the RHM maps included 10,000 to 18,000 spectra for a single slide. The exposure time for each pixel was 6 s. The pixels (step size) were 1 µm or smaller depending on the size of the area of interest, which varied from 80 µm × 80 µm to 140 µm × 140 µm.

### Raman measurements of isolated DNA

Raman spectra of two differently methylated commercially available genomic DNAs, both isolated from Jurkat cells (provided by Thermo Scientific), were acquired to explore reference Raman markers of DNA methylation. The supplier ensures a ≥ 98% CpG methylation level of CpG-methylated Jurkat genomic DNA. Spectra were collected with the same device described above. The acquisition time was 30 s per spectrum. Similarly, as tissue data, DNA spectra were acquired in the 1900–600 cm^−1^ range, with a spectral resolution of 2 cm^−1^.

### Pathological re-evaluation of areas of interest

After obtaining initial maps and basic spectral clustering, re-evaluation of the samples was possible. The same pathologists precisely selected and marked areas of cancer cells on the Raman map images. This step allowed nonrandom identification of valuable spectra, multivariate data analysis, and comparison. The good resolution of the collected hyperspectral maps made it possible to distinguish particular cancer cell elements, such as the nucleus and cytoplasm or the stroma compartment of the tumor.

### Multivariate data analysis

Data analysis was conducted in the MATLAB (RRID:SCR_001622) environment from MathWorks (Natick, USA). The preprocessing of acquired FTIR and Raman spectra involved sequentially applying a baseline correction (3rd polynomial order), smoothing with the Savitzky–Golay algorithm (third-order, 17 smoothing points), and normalization (Standard Normal Variate) in the range characteristic for biological molecules (1800–800 cm^−1^).

Infrared maps were processed with hierarchical cluster analysis (HCA) according to a previously described procedure [5].

The analysis of Raman data was divided into three parts related to the methods used. First, by the use of HCA (Ward’s method), the relationships between spectra (the matrix of spectra) were revealed. HCA allowed dividing spectra into clusters found in the dendrogram. The main advantage of using HCA is its ability to create false-color maps based on dendrograms, which illustrate the arrangement of clusters determined during the analysis [21]. HCA enabled direct comparison of the obtained experimental spectra. Based on the HCA results, the cancerous regions on false-color Raman maps were distinguished. Maps were analyzed independently; however, the same colors (revealed by the comparative analysis of clusters’ averaged spectra) were used to represent areas of similar chemical composition and structure in all of them. The second derivatives of the spectra from these sectors were calculated and utilized in subsequent principal component analysis (PCA). Because PCA makes it possible to reduce the dimensionality of the collected data, based on its results, we recognized the differences in Raman spectra from the cancerous regions for each studied type of PC tissue. During PCA, two important plots were produced, specifically the scores and loading plots. The first one enabled the separation of samples based on similarities, and the latter visualized differences in the spectra causing data separation marked as peaks. Scores and loading plots allowed us to identify areas of cancer. In parallel, on Raman maps, the nonnegative matrix factorization (NMF) method was carried out. NMF transformed the high-dimensional data into two matrices, which uncovered the meaningful features of the collected data. Most importantly, the NMF-generated matrices presented the chemical compounds of the spectra and allowed the production of false-color distribution maps. The nucleic acid components extracted in NMF analysis were compared across all studied PC types.

### CNN architecture

The last part of the study involved training CNN in the classification of raw Raman spectral data. The CNN was trained in predicting five classes, representing AVAC, IPMC, cPDAC, stroma/empty space, and benign pancreatic tissue. A total of 18 CNN-predicted map images were acquired for PC tissue from 14 patients, specifically 6 AVAC cases, 4 cPDAC cases, 6 IPMC cases, and 2 benign pancreatic tissue cases. We used a custom-designed CNN architecture with 18 convolutional layers for feature identification and 4 fully connected layers for classification. The programming of the CNN was performed in Python version 3.10.5 (IPython, RRID:SCR_001658) with TensorFlow (RRID:SCR_016345) and Keras application programming interfaces (API). A “sequential” base model was utilized. The details of the proposed CNN architecture are summarized in Supplementary Figure S1. For each layer, a “glorot uniform” initializing mode was used. The “Adam” optimizer and “categorical crossentropy” loss function were applied. The training involved 150 epochs with a batch size equal to 105. The selected spectra for each class were combined and shuffled randomly. The intensity values from each spectrum were combined with Raman shift values to form a 2D NumPy (NumPy, RRID:SCR_008633) array. The total number of spectra used for training/testing was 53,879 with the NumPy array shape presented as (53879, 2, 512) and included spectra from cancerous and noncancerous areas, as well as benign pancreatic tissue areas. Then, class arrays were “one-hot encoded,” and the training and testing dataset split was conducted with a 70/30 ratio. The approximate CNN training time was 45 min.

### CNN training and testing dataset selection

Data for training and initial testing of the CNN were selected on RHM maps plotted from raw (unprocessed) spectral data. To support the benefits of CNN-based automation, for CNN dataset creation, we did not use HCA maps but unstained optical microscopy tissue slide images, normally collected before Raman measurements. The MATLAB (RRID:SCR_001622) environment from MathWorks (Natick, USA) with the plot selection tool by John D'Errico was used to annotate the areas of cancer as separate classes (AVAC, IPMC, and cPDAC). Additionally, the stroma/empty class was annotated. The selections were performed by a pathologist experienced in PC and spectral data analysis. The tissue section containing the benign pancreas was used analogously for the creation of the benign class dataset. The process of training dataset annotation is depicted in Supplementary Figure S2.

### CNN-based classification plotting

In the last step of the CNN-based classification, the CNN was used to generate the prediction maps. Every spectrum obtained with RHM for each tissue sample was fed to the CNN and classified into one of the classes. The predicted class values (classes 0–4 standing for stroma/empty, AVAC, IPMC, cPDAC, and benign) created an array, which combined with the x and y coordinates of the original RHM map enabled the plotting of the CNN classification map image. Each image pixel expressed a CNN-predicted class with a different color. The plotting was performed in Python (IPython, RRID:SCR_001658) with the MatPlotLib library (MatPlotLib, RRID:SCR_008624). The total number of spectra used for generating the prediction maps was 211,355, of which 157,476 were new spectral data not trained on or tested by the CNN.

### CNN extracted features visualization plotting

To better understand the CNN workflow in spectral prediction, we visualized the features extracted by the CNN model in prediction mode. For each PC group, 5 representative spectra were processed. The outputs of the CNN at the beginning (dense layer with 512 neurons), middle (dense layer with 256 neurons), and end (dense layer with 128 neurons) of the classification process were drawn as linear plots. The NumPy (NumPy, RRID:SCR_008633) array shapes were (1, 512), (1, 256), and (1,128). The plotting was performed in Python (IPython, RRID:SCR_001658) with the MatPlotLib library (MatPlotLib, RRID:SCR_008624).

## Results

In the first step of the study, we applied infrared imaging to reveal chemical differences in various types of PC, specifically AVAC, IPMC, and cPDAC (“FTIR imaging of PC types” section). Proper interpretation of the spectral differences within the dataset requires the use of multivariate data analysis, which permits reducing the dimensionality of data and obtaining information on the structure of the dataset, spectral similarity, and variability. HCA was used to distinguish regions of tissues with different spectral characteristics related to the presence and concentration of various biochemical components, such as proteins and nucleic acids [5].

To precisely reveal chemical variability among pancreatic malignancies (AVAC, cPDAC, and IPMC), we implemented Raman imaging (“RHM mapping of pancreatic tumors” section). On the one hand, Raman maps are relatively small compared to FTIR maps, thus, they represent spectra obtained from a limited number of cancerous cells or areas of cancer stroma. On the other hand, RHM provides submicrometric spatial resolution enabling the acquisition of signals from subcellular components, such as cell nuclei or cytoplasm, and allows separate and detailed investigations of those regions [5], which was required by our study. To obtain good-quality RHM maps, the measured tissue should be spectroscopically adequate. This is best verified on a larger scale. Although the resolution of infrared mapping is restricted by the diffraction limit related to infrared wavelength, we used FTIR spectroscopy as a supportive tool to obtain such large-scale maps. This important step allowed the selection of spectroscopically representative areas for further investigation by Raman mapping. The FTIR signal from the preselected areas (preselected by pathologists on tissue slide images—see “Tissue slide preparation” in the “Methods” section) of tissue sections was typical, demonstrating the presence of functional groups expected to be found in tissues (purified from paraffin). Additionally, FTIR data confirmed that the thickness of tissues was homogeneous or that tissues were properly attached to the substrate and free from contamination.

For a selected region of each PC tissue sample, we performed RHM with a pixel size of 1 µm. The obtained RHM maps of PC tissues were treated with the HCA algorithm, which permitted the division of Raman spectra into clusters. Specifically, each studied Raman map of PC was divided into seven spectroscopically different clusters [21]. Furthermore, to comprehensively investigate the molecular relations of different components, all RHM maps were analyzed using NMF algorithms. NMF highlights the main components of Raman spectra from the collected maps. A significant advantage of the NMF method is its ability to extract the signal from water as one of the components. Thus, a detailed analysis of Raman signals from other components (proteins or nucleic acids) without the disruption of overlapping water bands is possible. Moreover, this ability is crucial when analyzing the secondary structure of proteins, where the O–H bending motion at 1643 cm^−1^ overlaps with the amide I band. Each of the derived NMF components corresponds to differences in the chemical composition of particular areas of the tissue.

The HCA allowed localization of the nuclei of the cancer cells, enabling us to further investigate these. The results of the PCA for each PC type are described in the “PCA of spectral data acquired from nuclei of the PC cells” section. To complete the analysis of PC nuclei, we present the results of NMF analysis for this region of the studied samples in the “Comparison of Raman signals from the nuclei of AVAC, IPMC, and cPDAC” section.

Finally, in the “Generating CNN-based prediction maps for AVAC, cPDAC, and IPMC” section, the CNN training and the CNN-prediction map generation results are summarized. Notably, in the “Visualizing the CNN extracted features” section, we briefly show the visualization of the CNN analysis.

### FTIR imaging of PC types

**Fig. 1:**
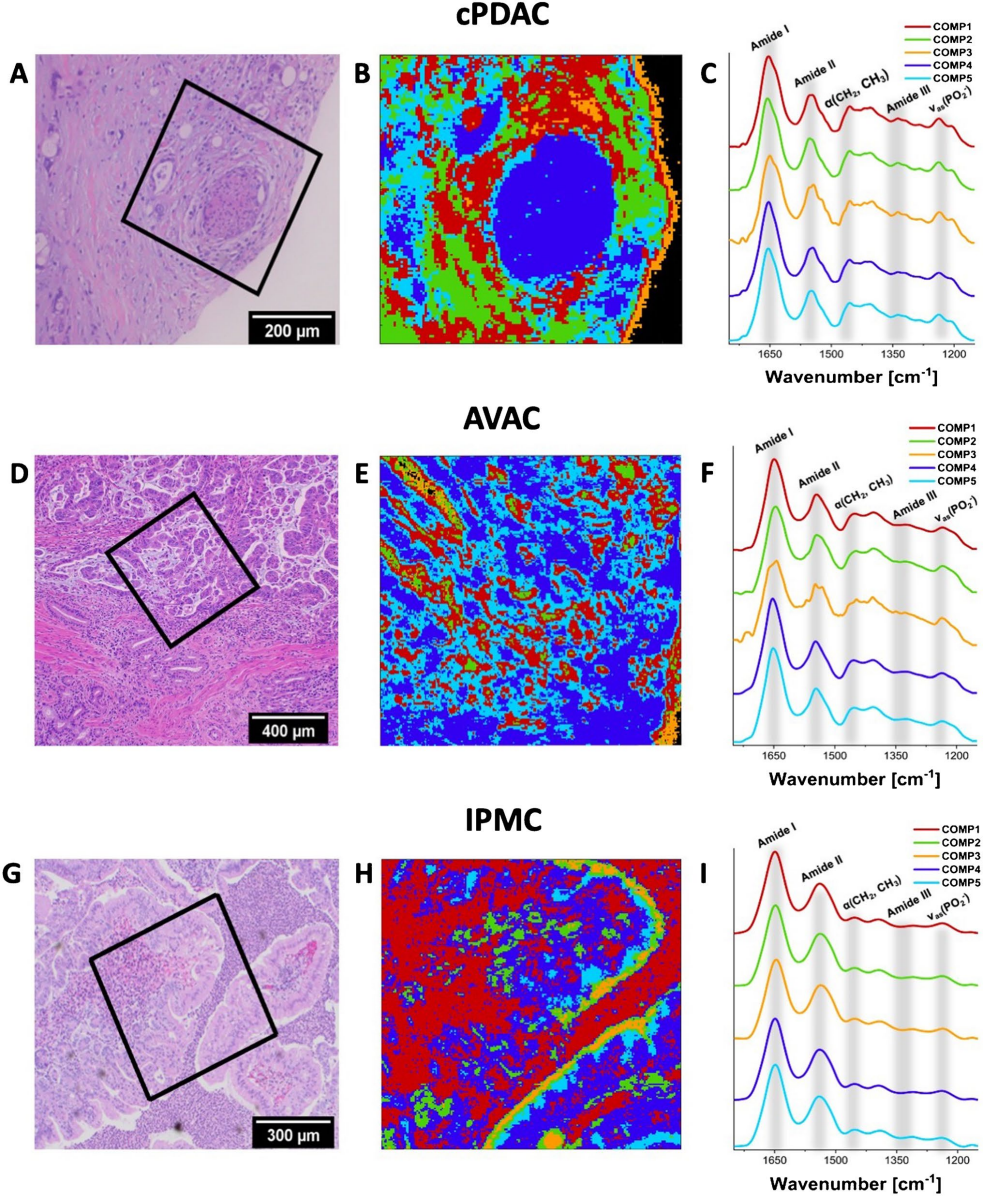
HCA results of infrared tissue maps of PC tumors. **A**, **D**, **G** Optical images of PC tissues stained with hematoxylin–eosin and marked areas of infrared map acquisition (H&E stain, original magnification × 100), **B**, **E**, **H** corresponding HCA maps, and **C**,** F**,** I** mean spectra of each cluster with marked characteristic protein and nucleic acid bands. The colors of the spectra correspond to the colors of the clusters in the HCA maps. The areas marked in black on the HCA maps of cPDAC and AVAC (**B** and **E**) correspond to a lack of tissue. Component 3 on HCA maps of cPDAC (**B**) corresponds to a border region of the tissue sample. See the main text (see “FTIR imaging of PC types” in the “Results” section) for interpretation

Representative results of FTIR imaging of PC tumors are presented in Fig. 1. The distribution of proteins and nucleic acids concentration is depicted in Supplementary Figure S3. For all the analyzed samples, 5 HCA components were revealed. The spectrally similar components are drawn with the same color.

The relatively intense band from the phosphate asymmetric stretching at 1230 cm^−1^ can be seen in component 1 (red spectrum). This component is associated with a high content of nucleic acids. We observed a relatively high abundance of component 1 in the investigated area of IPMC tissue, suggesting a high content of cells in comparison with the protein-rich extracellular matrix. The richness of nucleic acids has been reported as typical for aggressive (highly metastatic) cancer cells [22]. However, in the IPMC sample (Fig. 1G and H), most of component 1 was composed of inflammatory cells (neutrophils and lymphocytes) that surrounded the cancerous glands. Interestingly, in the spectra acquired from cPDAC and AVAC tissues, the asymmetric stretching of phosphate splits into two. This splitting confirms the presence of hydrogen bonding to oxygen atoms of phosphate groups observed previously in malignant colorectal cancer [23]. Conversely, in a sample of IPMC tissue, only one peak from phosphate is recognized, similar to that reported in normal (benign) colon tissue [23]. Component 2 (green spectra), which was observed mainly in cPDAC, appears in the locations of the tissue with high band area ratios of 1450/1650 cm^−1^ and 1406/1650 cm^−1^. These bands correspond to the bending motions of the CH_3_ groups in protein side chains (1450, 1406 cm^−1^) and to the amide I band (ν(C = O) and δ(N–H)) from cellular proteins (1650 cm^−1^) [24, 25]. The observed high ratios may be considered a hallmark of collagen accumulation [26] in the dense fibrotic stroma of ductal adenocarcinoma [27]. Since the spectra of component 3 are located mainly on the boundaries of the tissue sections, it likely was explored by HCA due to the different thickness of the sample rather than its chemical composition, and therefore detailed interpretation of this component was not performed. No significant differences in the abundance and distribution of component 3 were observed for all investigated PC tissue sections. High-intensity amide I and amide II (δ(N–H) and ν(C-N)) bands at 1650 cm^−1^ and 1550 cm^−1^, respectively, can be observed in component 4 and component 5. Both correspond to a high cellular protein ratio since the observed ratios of the bending modes of aliphatic chains in proteins concerning amide I (1450/1650 cm^−1^ and 1406/1650 cm^−1^) are low in comparison with the ratios calculated for the spectra of component 2. A high content of this component is observed in tissue samples of AVAC. The second derivatives of each component, demonstrating spectral differences between them, are presented in Supplementary Figure S4.

### RHM mapping of pancreatic tumors

**Fig. 2:**
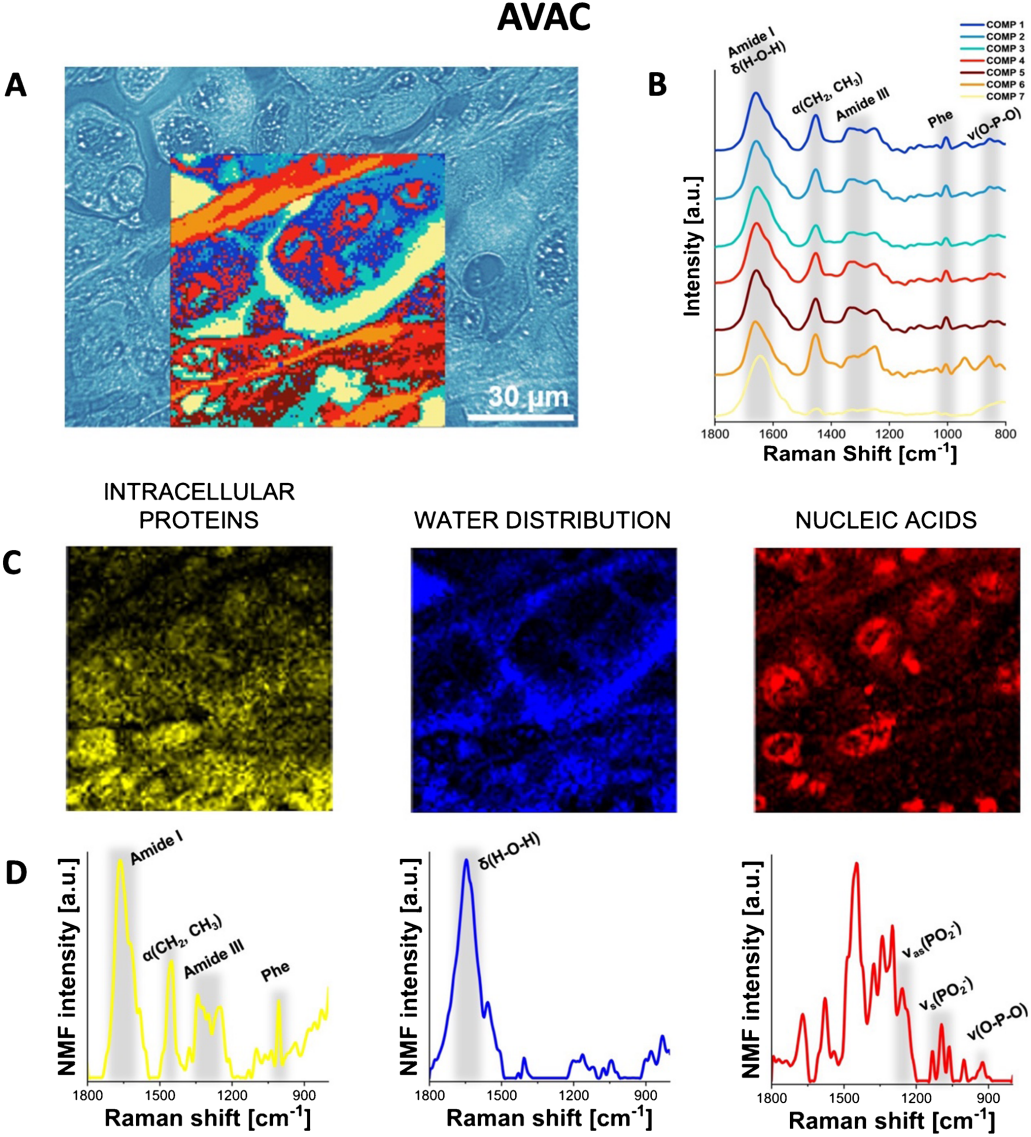
Raman molecular imaging of AVAC tissue. **A** The optical unstained tissue image with superimposed HCA map (unstained tissue, original magnification × 600), **B** mean Raman spectra from the HCA (the color of each spectrum corresponds to the color of the cluster in the HCA map), **C** distribution of the NMF components, highlighting proteins, water, and nucleic acids, and **D** corresponding plots of these NMF components. See the main text (see “RHM mapping of pancreatic tumors” in the “Results” section) for interpretation. For the comparison of HCA, unstained, and hematoxylin and eosin-stained slides, see Supplementary Figure S5

**Fig. 3:**
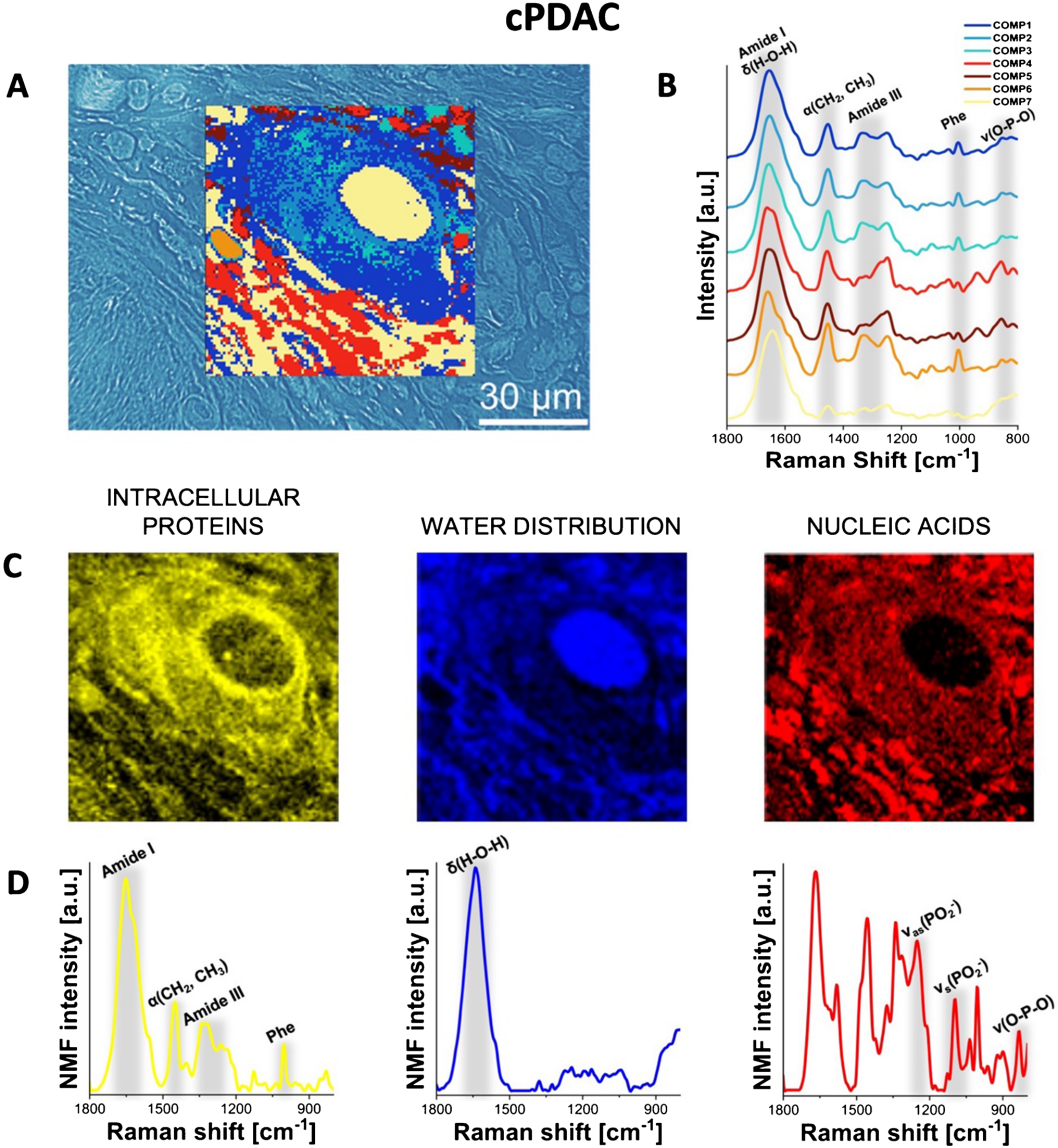
Raman molecular imaging of cPDAC tissue. **A** The optical unstained tissue image with superimposed HCA map (unstained tissue, original magnification × 600), **B** mean Raman spectra from the HCA (the color of each spectrum corresponds to the color of the cluster in the HCA map), **C** distribution of the NMF components, highlighting proteins, water, and nucleic acids, and **D** corresponding plots of these NMF components. See the main text (see “RHM mapping of pancreatic tumors” in the “Results” section) for interpretation. For the comparison of HCA, unstained, and hematoxylin and eosin-stained slides, see Supplementary Figure S5

**Fig. 4:**
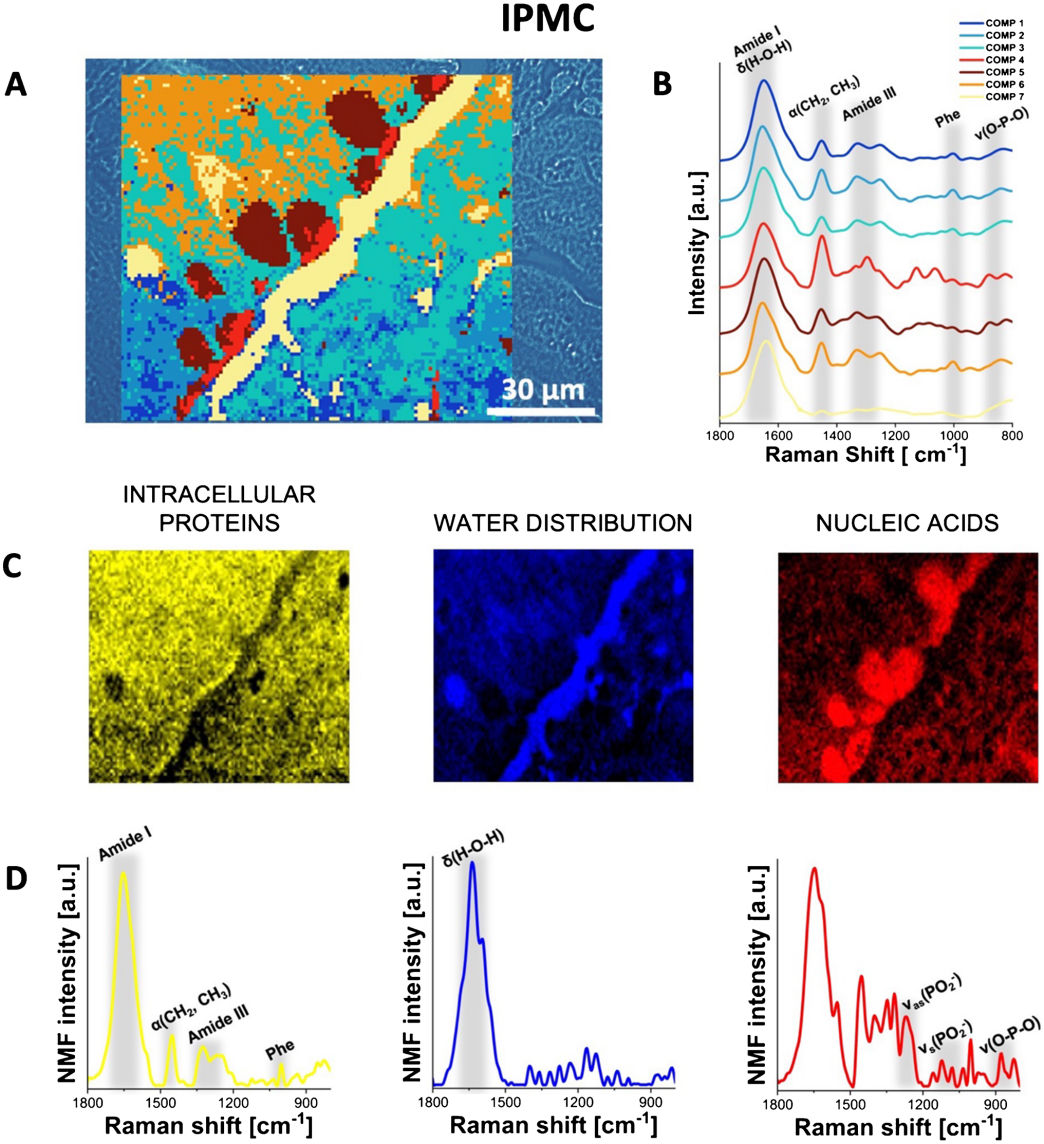
Raman molecular imaging of IPMC tissue. **A** The optical unstained tissue image with superimposed HCA map (unstained tissue, original magnification × 600), **B** mean Raman spectra from the HCA (the color of each spectrum corresponds to the color of the cluster in the HCA map), **C** distribution of the NMF components, highlighting proteins, water, and nucleic acids, and **D** corresponding plots of these NMF components. See the main text (see “RHM mapping of pancreatic tumors” in the “Results” section) for interpretation. For the comparison of HCA, unstained, and hematoxylin and eosin-stained slides, see Supplementary Figure S5

The results of HCA of the typical tissue sections of AVAC, cPDAC, and IPMC are shown in Figs. 2, 3, and 4, respectively. The spectroscopically similar HCA components in each presented map are marked by the same colors. In the figures, the HCA components 1–3 (blue colors) are characterized by prominent bands characteristic of proteins, specifically the CH_2_ and CH_3_ bending motions at 1450 cm^−1^, the amide III in the range of 1350–1220 cm^−1^, and phenylalanine (Phe) at 1000 cm^−1^ (for detailed band assignments, see Supplementary Table S2). No significant spectroscopic differences, such as changes in peak ratios and shifts of peak spectral positions, were detected between these three components. They differed mainly in total intensity, which corresponds to the density of the tissue material and the thickness of the sample. These components specify the location of the cytoplasm of PC cells. In the spectra of components 4 (red spectra) and 5 (purple spectra), the band at 1370–1340 cm^−1^, attributed to DNA bases (T, A, G) [28] and phosphate motions at 1230 cm^−1^ and 1080 cm^−1^, are well pronounced and indicate the location of cellular nuclei. The relatively high intensity of bands attributed to proteins, together with the pronounced bands at 1250 cm^−1^ and 940 cm^−1^ in component 6 (orange spectra), indicate the presence of collagen fibers in the extracellular matrix [29]. The spectrum of component 7 (cream color), which is dominated by the O–H bending motion from water, represents areas lacking tissue material.

Our results obtained with RHM combined with HCA accurately confirmed the locations of cellular components previously assumed based on unstained optical microscopy slide images, separating the areas of the cytoplasm and nuclei of cancer cells. Moreover, the comparison of spectroscopic band intensity revealed significant differences among the three types of PC tumors, specifically AVAC, IPMC, and cPDAC. Based on the spatial distributions of components 4 and 5, we estimated the relative amount of nucleic acids and the size of cellular nuclei. A significant difference was observed in the distribution of component 6 (orange color), indicating the presence of fibrillar proteins such as collagen. In all investigated samples, component 6 is located in the extracellular matrix; however, in cPDAC and AVAC, it forms aggregates/fibers, while in the cross-section of IPMC, it is distributed more homogeneously. The homogeneous distribution of components 1–3 was detected in all the investigated types of PC, as expected, since these components are dominated by bands from proteins and peptides. These compounds were found lining the pancreatic duct in cPDAC.

**Fig. 5:**
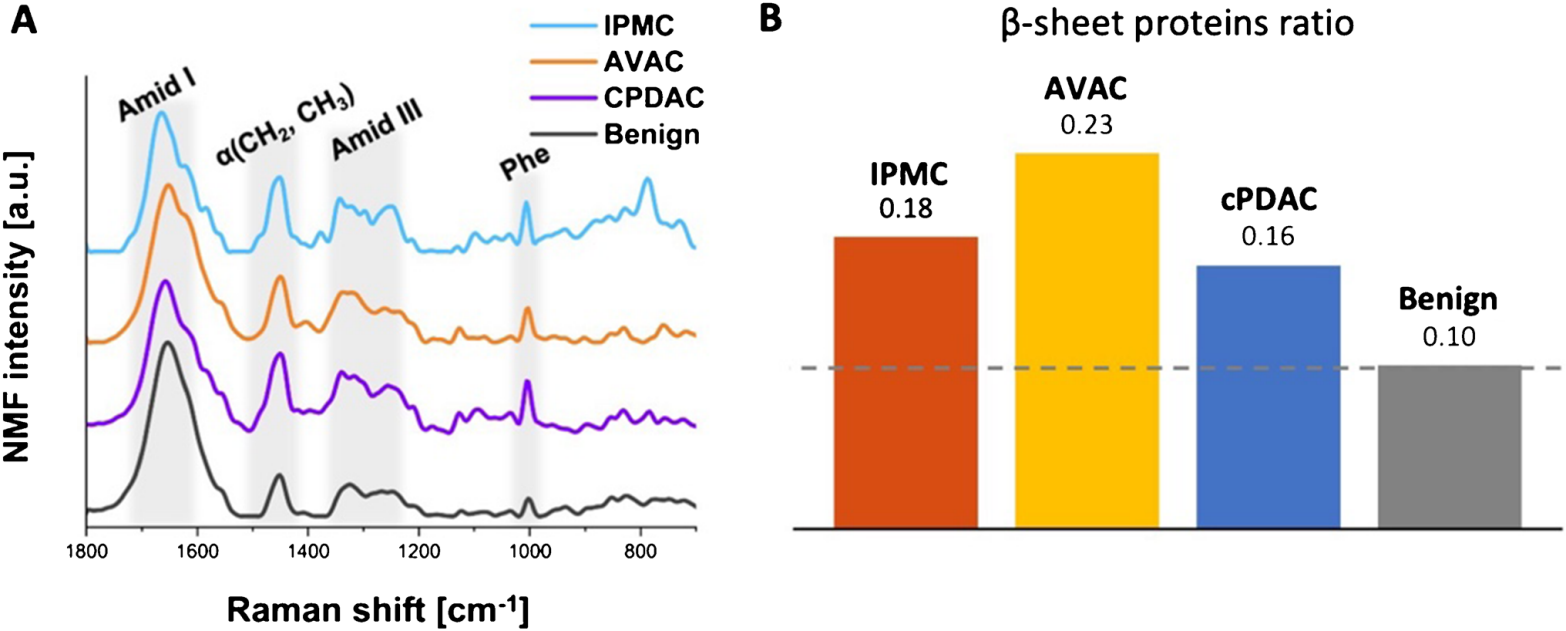
Spectral marker bands of proteins in NMF components of all studied PC types and benign pancreatic duct tissue. **A** A comparison plot of NMF components and **B** the relation between Raman bands characteristic of proteins’ β-sheet secondary structure (1690–1668 cm ^−^^1^, β-sheet amide I to 1750–1514 cm ^−^^1^, total amide I)

NMF analysis was used to discover specific features of the studied RHM maps that formed a unique “fingerprint” for each type of PC. We present the three main components of the spectra obtained from individual PC tissues, visualized as NMF maps. Figures 2, 3, and 4 depict the NMF components of the Raman spectra from acquired RHM maps, calculated separately for each PC type, specifically AVAC (Fig. 2C and D), cPDAC (Fig. 3C and D), and IPMC (Fig. 4C and D). The NMF components and corresponding NMF maps of benign pancreatic ductal tissue are presented in Supplementary Figure S6. Each component is associated with a characteristic chemical composition, including intracellular proteins, water, and nucleic acids, that represents the particular region of the studied tissue. The lipid component was not analyzed, because the lipids were dissolved by xylene/ethanol treatment in the slide preprocessing routine before RHM map acquisition. In the figures, the first NMF component associated with intracellular proteins is characterized by high intensities of protein bands that include Phe vibrations at 1004 cm^−1^, amide I vibrations at 1700–1600 cm^−1^, or amide II vibrations at 1580–1480 cm^−1^ [24, 25]. The second NMF component matches OH bending vibrations at 1643 cm^−1^, which correlates with the water distribution, and the NMF component of nucleic acids demonstrates the higher intensity of bands assigned to the stretching of phosphate groups from the DNA backbone at 1090 cm^−1^ and 1256 cm^−1^ (Figs. 2D, 3D, and 4D) [30]. The comparison of the second derivatives of NMF components related to proteins is presented in Supplementary Figure S7. The redshift (toward lower energy) of the amide I band from 1650 (IPMC and cPDAC) to 1664 cm^−1^ (AVAC) illustrates the relatively high content of the β-sheet secondary structure in AVAC [31], presumably due to the presence of β-sheet-rich proteins. In Fig. 5A, characteristic spectral bands of proteins are seen, and the relative ratio of β-sheet proteins is depicted, showing a high prevalence in all studied PC subtypes (Fig. 5B). Specifically, the relation between Raman bands characteristic of proteins’ β-sheet secondary structure, that is, the β-sheet amide I (1690–1668 cm^−1^) to total amide I (1750–1514 cm^−1^), was 0.16, 0.23, and 0.18 for cPDAC, AVAC, and IPMC, respectively, whereas for benign pancreatic duct control, this ratio was 0.10.

### PCA of spectral data acquired from nuclei of the PC cells

**Fig. 6:**
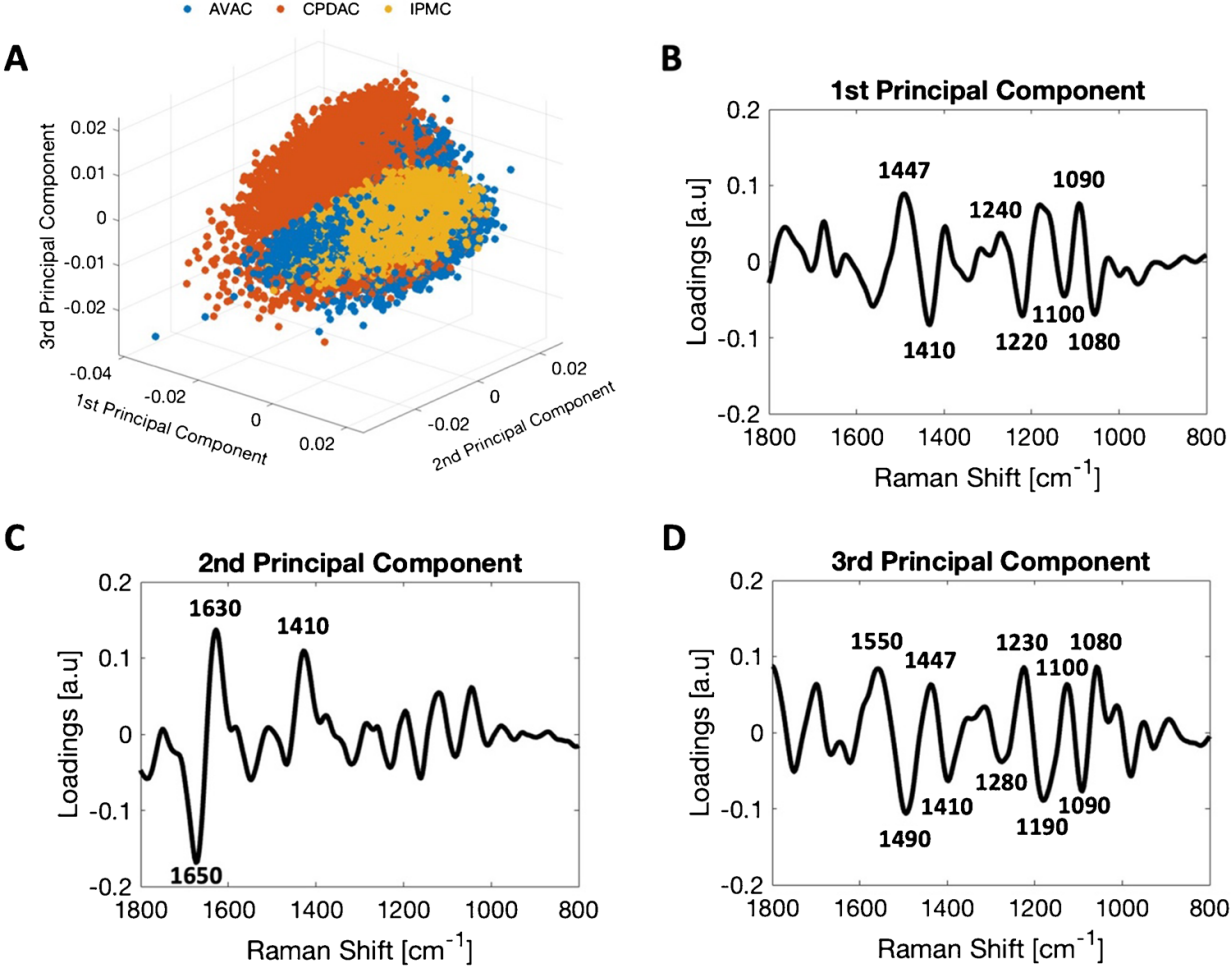
PCA of spectra extracted from cancerous cell nuclei from all the investigated tissue sections. **A** PCA plot showing all three principal components. PC-1 explains 44%, PC-2 explains 25%, and PC-3 explains 12% of the total variance within the dataset. A clear clustering of cPDAC, IPMC, and AVAC along PC-1 is distinguishable (please note that points representing AVAC are partially covered by the IPMC points). Additional pairwise comparison 3D and 2D scores plots are presented in Supplementary Figure S8. **B** Loadings plots of PC-1, **C** PC-2, and **D** PC-3. See the main text (see “PCA of spectral data acquired from nuclei of the cancer cells” in the “Results” section) for interpretation

**Fig. 7:**
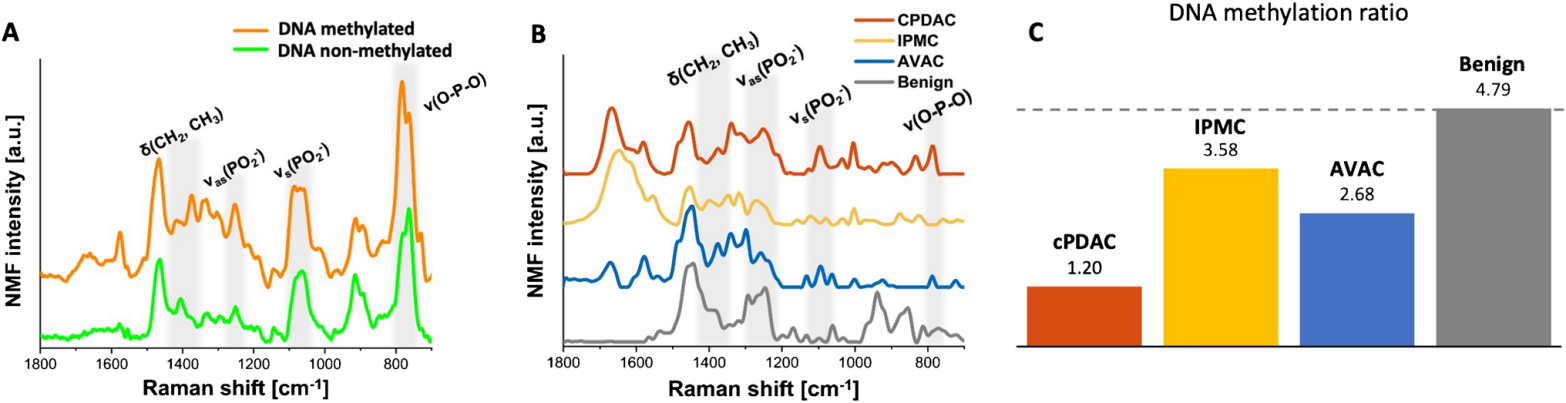
Spectral marker bands of methylation and DNA conformation in NMF components corresponding to chromatin (nucleic acids and histone proteins). **A** Raman spectra acquired from reference isolated samples of methylated and unmethylated DNA with highlighted characteristic spectral bands, **B** a comparison plot of NMF components calculated for chromatin of all studied PC types and a benign pancreatic duct tissue, and **C** the relation between Raman bands characteristic of DNA methylation in mean NMF components of each of the studied PC types and the control benign pancreatic duct tissue (1360–1420 cm^−1^, δ(CH2, CH3) to 1050–1150 cm^−1^, ν_s_(PO_2_.^−^)). The comparison of second derivatives of NMF components related to DNA conformation is presented in Supplementary Figure S9

PCA was applied to detect similarities within the spectral datasets acquired from cancer cell nuclei of PC tumors of different origins (AVAC, cPDAC, and IPMC). Each spectrum is presented as an individual point in the space of the new, orthogonal variables called principal components, as demonstrated by the scores plot in Fig. 6A. This plot is best viewed virtually in all 3 dimensions, as in the plain representation image of the 3D plot, the relationship between some points is slightly obscured by other points. For clarity, 2D scores plots are presented in Supplementary Figure S8. To avoid the influence of the baseline, calculations were performed on the second derivatives of the spectra. Corresponding loading plots highlighted the bands determining the separation along each principal component. However, since we analyzed second derivatives, the observed maxima were characteristic of the spectra located on the negative sides of the corresponding principal component (scores plot), whereas the minima highlighted bands present in the spectra at the positive side of the principal component. In Fig. 6A, the spectra collected from cPDAC are clustered on the positive side of PC-1, in contrast to the spectra of AVAC and IPMC, which are located on the negative side of PC-1. The loading plot corresponding to PC-1 (Fig. 6B) is dominated by bands from the phosphodiester contributions of nucleic acids in the spectral range 1240–1080 cm^−1^, as well as methyl and methylene deformational motions at 1447 cm^−1^ and 1410 cm^−1^. The presence of the band from CH_3_ wagging and bending at 1410 cm^−1^ and the spectral position of phosphate stretching at 1100 cm^−1^ are observed on the positive side of PC-1, indicating more methylated DNA in the A conformation [28, 32] in AVAC and IPMC compared to cPDAC. Specifically, the relation between Raman bands characteristic of DNA methylation, that is, 1360–1420 cm^−1^ (δ(CH2, CH3)) to 1050–1150 cm^−1^ (ν_s_(PO_2_^−^)), was 1.20, 2.68, and 3.58 for cPDAC, AVAC, and IPMC, respectively, whereas for the benign pancreatic duct control, this ratio was 4.79 (Fig. 7C). The separation along the 2nd principal component (PC-2) in Fig. 6A is mainly driven by the secondary structure of nuclear proteins and peptides but also by the methylation level of DNA, as presented in the loading plot of PC-2 (Fig. 6C). PC-2 is dominated by amide I at 1630 cm^−1^ (β-sheet) and 1650 cm^−1^ (α-helix, turns, unstructured coils), but also CH_3_ wagging and bending motions at 1410 cm^−1^. The negative correlation of PC-2 loading at 1650 cm^−1^ indicates a relatively high content of secondary structures in proteins and peptides located within nuclei of IPMC, such as α-helices, turns, and unstructured coils. No clear separation of spectra acquired from AVAC and cPDAC throughout PC-2 is observed. Similar to PC-1, clustering along PC-3 (Fig. 6A) is related to the spectral changes in the bands from methyl and methylene motions, as much as bands of phosphate groups from nucleic acids.

### Comparison of Raman signals from the nuclei of AVAC, IPMC, and cPDAC

In Raman spectra, molecular modifications assigned to DNA methylation and related conformational rearrangements are observed as changes in the intensities and spectral positions of particular bands. As shown in Fig. 7A, the increased intensity of the Raman signal in the region of 1410–1360 cm^−1^ indicates the methylation process [33]. Furthermore, a shift toward lower energy of the bands that reflect the symmetric (~ 1090 cm^−1^) and asymmetric stretching (~ 1256 cm^−1^) of phosphate is observed in the spectrum of methylated DNA, indicating a partial conformation change from B-like DNA to A-like DNA [34]. By analyzing the NMF components corresponding to the nucleic acids and proteins in cancer cells presented for each PC sample, we noticed that the changes characteristic of the methylation process were clearly visible in the spectra acquired from IPMC tissue (Fig. 7B). Additionally, the appearance of a new band associated with the methylation process can be observed at 1408 cm^−1^ along with the shift of bands corresponding to the DNA backbone vibrations. Specifically, the band observed at 1090 cm^−1^ (symmetric stretching of phosphate groups from DNA backbone) was split into two peaks, and similar to the band observed at 1256 cm^−1^ (asymmetric stretching of phosphate groups), it was slightly shifted toward higher wavenumbers. These changes can be attributed to conformational modifications of DNA, followed by the DNA methylation process [34]. The intensity changes of cytosine Raman bands from the N–H bending at 1575 cm^−1^, along with the NH_2_ bending and C4–NH_2_ stretching at 1614 cm^−1^, depicted in Fig. 7A, were associated with DNA methylation of isolated single-stranded DNA [35]. However, the straightforward observation of such spectral differences in NMF components, representing cellular nuclei of cancerous tissues (Fig. 7B), is largely prohibited due to the overlapping amide I band from histone proteins, thus we did not include these in the analyses. The full description of the Raman bands is presented in Supplementary Table S2.

### Generating CNN-based prediction maps for AVAC, cPDAC, and IPMC

**Fig. 8:**
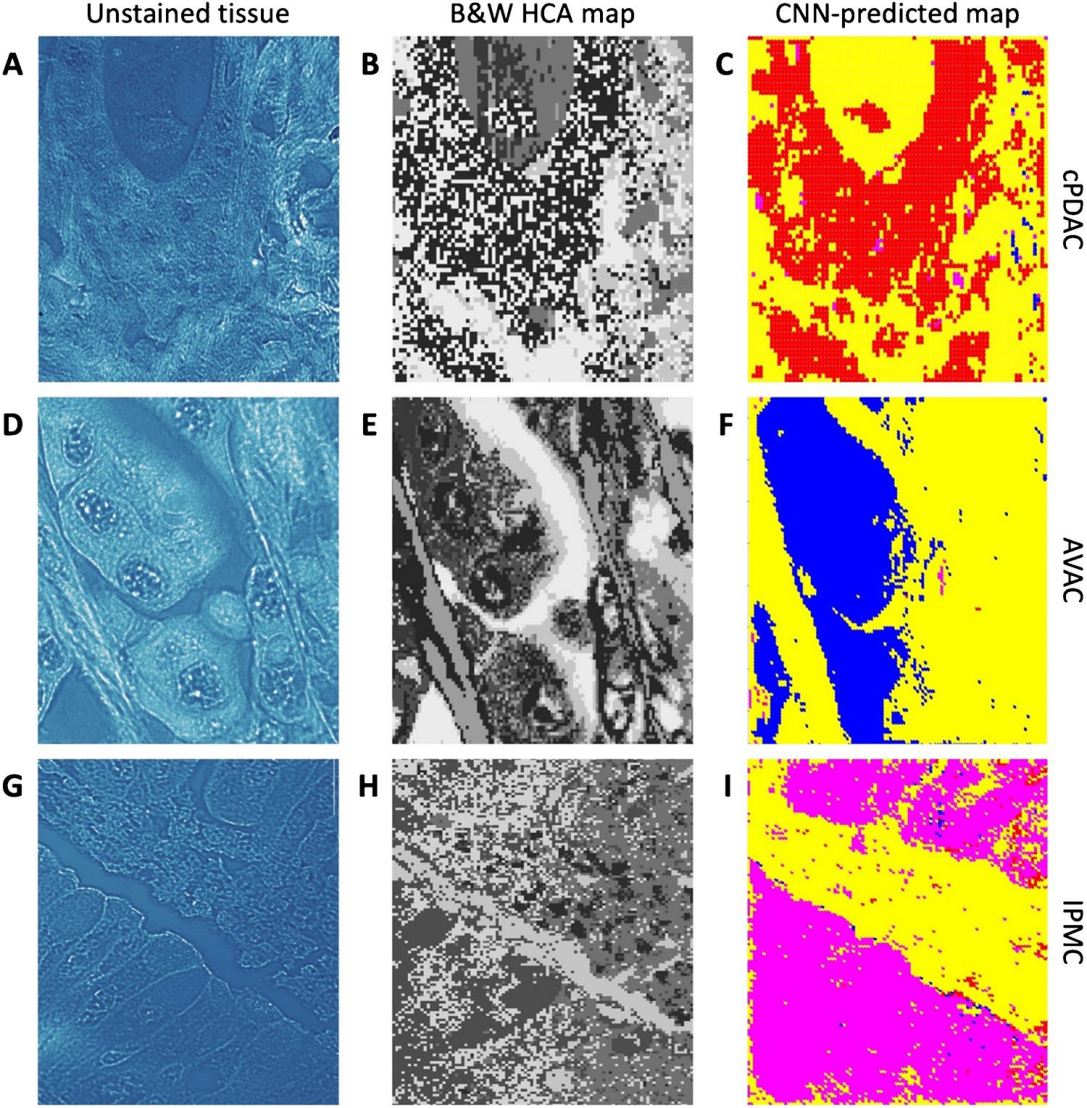
Comparison of HCA and CNN-predicted maps of PC tumors. **A**, **B**, **C** Unstained, optical microscopy slide images of cPDAC, AVAC, and IPMC, respectively (unstained tissues, original magnification × 600). **B**, **E**, **H** Black and white HCA maps of cPDAC, AVAC, and IPMC, respectively. **C**, **F**, **I** Prediction map images generated by the CNN, which classified each PC tumor of different origins with pixel colors, specifically red for cPDAC, blue for AVAC, and magenta for IPMC. Yellow was used to mark the stroma/empty class, and green (not shown in this figure) was used to mark benign pancreatic tissue

On each PC tissue map, a pathologist experienced in PC and spectral data analysis marked cancerous and noncancerous areas (see “CNN training and testing dataset selection” in the “Methods” section for details of the methodology) (Supplementary Figure S2). The annotation process was repeated for each PC type (AVAC, cPDAC, IPMC) and the benign pancreatic tissue areas in a single sample. Then, CNN training was conducted. The CNN was trained to classify spectra from all RHM maps into 5 classes, specifically to predict whether these were AVAC, cPDAC, IPMC, benign tissue, or stroma. The accuracy achieved by the CNN in the training process was slightly over 99%, with a validation accuracy equal to 94% (validation loss of 0.17). The next step was employing the trained CNN model to generate prediction maps based on the spectra at each pixel of the original raw RHM map. The same CNN was used for predicting all 5 classes. The exemplary resulting maps of PC tissues are presented in Fig. 8. CNN handled the distinction of the origin of PC tumors efficiently. Moreover, the obtained maps were comparable to human-dependent HCA maps, which are time-consuming to prepare. All maps generated with the CNN may be found in Supplementary Figures S10, S11, S12, and S13.

### Visualizing the CNN extracted features

After successfully plotting the CNN-predicted maps, we tried to better understand the mechanisms of the CNN feature extraction. For each PC type, we visualized the CNN outputs as linear plots. In this way, we could see what the spectral features specific for AVAC, cPDAC, and IPMC looked like for the CNN. The plots of exemplary spectra obtained from the cancerous regions of each PC tissue are depicted in Supplementary Figures S14, S15, and S16.

## Discussion

This study’s main goal was to demonstrate that RHM differentiates between three major groups of pancreatic masses, specifically cPDAC, IPMC, and AVAC. This point was established twofold by the identification of spectroscopic differences and the usage of neural networks.

Multiple studies have used RS methods for the detection of various cancers, but most of them rely on random blind spot measurements [36] or rare grid mapping [37]. We used a more comprehensive and systemic approach, which involves collecting high-resolution RHM, followed by a selection of areas of interest in acquired resulting maps, and then proceeding to the analysis. The use of Raman imaging for PC subtyping was not described before, except in a recent preliminary report [5]. A single study by other authors utilized hyperspectral mapping of PC tissue samples; however, they used FTIR spectroscopy for spectral data acquisition [26]. Although FTIR can be used to classify PC tissues, the RHM allowed deeper insight into cancerous tissues and the extraction of detailed information about the local heterogeneity that could not have been achieved with IR-based methodology due to the physical limitations of the latter, such as the diffraction limit. Nevertheless, our study started with FTIR microspectroscopy as well, although only as an initial step. This allowed us to view the distribution of tissue components on a larger scale, recognizing their spatial heterogeneity. FTIR permitted analysis of the tumor stroma compartment and selection of the areas of interest for subsequent Raman measurements. Moreover, FTIR analyses carried out for studied PC samples showed characteristic features of PC stroma, such as high collagen accumulations reported in [26, 27]. Subsequently, RHM investigations confirmed the collagen distribution surrounding the malignant epithelium in PC [38] and showed differences in collagen arrangement patterns between cPDAC, AVAC, and IPMC. RHM allowed spectral analyses of nuclear and cytoplasmic regions of PC cells separately. This was not possible with FTIR only, because of its lower resolution [5]. Consequently, we separated the spectral components of PC tissues into water, proteins, and nucleic acids with NMF. This approach facilitated the analysis of DNA methylation patterns and the secondary structure of proteins and revealed the differences among the studied PC types.

The global DNA methylation status of AVAC and IPMC or cPDAC has not been compared thus far. DNA methylation is one of the main epigenetic modifications of many malignancies. The methylation patterns of numerous PC-related genes have been well documented for cPDAC [39, 40], AVAC [40, 41], and IPMC [39, 40]. Recognizing some of these patterns can help predict PC aggressiveness or the survival rates of PC patients [42]. A downside of the genetic-based methodology in the DNA methylation status assessment is that the results highlight only single (or multiple, but still individual) gene promoter modifications. The “global view” is lost when looking too deeply or too specifically. Conversely, in VS, the information obtained from the analyzed sample is holistic and allows “fingerprint-like” interpretation. The ability to detect the DNA methylation status was established by the use of VS techniques such as surface-enhanced Raman spectroscopy (SERS) [43] or attenuated total reflection Fourier transform infrared spectroscopy (ATR-FTIR) [44]. However, most of the mentioned studies have not evaluated tissue samples but isolated DNA strands or cells.

In our study, the chosen approach of RHM allowed detailed analysis of the spectra from PC cellular nuclei. Combined with NMF and PCA techniques, we recognized DNA methylation patterns in cPDAC, IPMC, and AVAC. The main DNA methylation aberrations recognized in cancerous cells are global hypomethylation and CpG island hypermethylation specific for particular genes [39]. Single-gene hypermethylation is responsible for PC carcinogenesis and progression [45]. Our findings confirm the global hypomethylation status of DNA in all PC types and are in line with the results described by other authors that cPDAC is associated with global hypomethylation [46]. However, it was found that the DNA of IPMN (the benign precursor of IPMC) was not hypomethylated [47]. Our results indicate that even though cancer arising from IPMN is slightly hypomethylated compared to benign pancreatic duct tissue, the DNA methylation ratio is highest among the other two PC types (AVAC and cPDAC). This might confirm that the DNA hypomethylation process is the domain of malignancy. Furthermore, the status of DNA methylation significantly differed between the studied PC groups (Fig. 7C). We are the first to compare the global DNA methylation levels among PC subtypes.

Circular dichroism (CD) spectroscopy is an efficient tool for determining the secondary structure of proteins in solutions [48]. For tissue samples in a solid state, however, Raman spectroscopy is far more effective [31]. In a recent work by Rasuleva et al. [49], the authors studied extracellular vesicles (EVs) derived from PDAC cell lines through CD spectroscopy and found significantly higher amounts of β-sheet proteins in PDAC cells and EVs than in nonmalignant samples. The authors suggested that the β-sheet-richness of proteins might be related to the activity of the mitochondria in malignant cells [50] and that it demonstrates the Warburg effect in PC [51]. Numerous β-sheet-rich proteins and peptides were reported to be critically involved in signaling pathways driving PC deregulation and progression, such as the SH2 and SH3 domains of the c-Src protein, epidermal growth factor (EGF), or the Hedgehog pathway proteins [52]. Furthermore, carcinogenesis is characterized by abnormal aggregation of proteins [53], meaning that proteins are misfolded into inactive amyloid fibers. For example, mutant p53, a product of the tumor suppressor gene that is mutated in over 50% of human malignancies, undergoes numerous aggregations in the course of tumor progression, such as metastatic ability or chemoresistance [54]. Further studies showed that the β-sheet protein secondary structure impaired the folding rates and promoted aggregation of these proteins [55].

Recognizing protein secondary structure in synchrotron radiation-based FTIR was a subject of the classification of human glial tumors [56]; however, no studies have investigated spectral differences in protein composition among PC subtypes. In our study, the analysis of NMF components associated with proteins of RHM spectra allowed us to determine the secondary structure of proteins in PC groups. We recognized the overall protein β-sheet richness. Additionally, their contents varied among the studied PC types and were particularly high in AVAC (Fig. 5B).

After successfully identifying spectroscopic markers specific to various PC groups using multivariate data analysis techniques, we trained a CNN in PC-type classification. The CNN-based approach has great advantages over standard processing. CNN is very sensitive to seemingly irrelevant differences in spectral data, requires no preprocessing, and permits automation. The idea of using CNN in spectral data classification is not new; however, none of the reported studies has utilized neural networks for the generation of PC tissue maps based on RHM data prediction. Li et al. [36] employed CNN to distinguish the Raman spectra of PC *vs.* benign pancreas and achieved results of 95% accuracy. In our study, we aimed at CNN-based PC subtyping, as the detection of PC *vs.* benign is possible with standard histopathological assessment and does not require sophisticated measurements. Furthermore, we examined over 211,000 spectra obtained from PC tissue samples, whereas the authors of the aforementioned article evaluated slightly over 2500 Raman spectra from the tumor incubated in the mouse vector. Our results in the CNN prediction of PC subtypes reached over 94%.

Multiple studies have described different CNN architectures in spectral data classification [57, 58]. Some of them used very deep and complex networks [59]. In contrast, we observed that the performance of the CNN is better when a simpler CNN architecture is used. If we added more layers to the CNN, it showed overfitting earlier. CNN is overfitting when it performs well with the training data but performs poorly with new datasets. Usually, when designing the CNN, one wants to prevent overfitting and grow the CNN’s potential to “generalize,” which stands for the ability to interpret new data well. The spectra from the RHM proved to be quite a simple model for the CNN; thus, it did not require very deep layers. Moreover, the CNN was trained and validated on only 25% of the RHM spectral data ultimately used for CNN-prediction map generation (53,879 of 211,355 spectra) and generalized efficiently on the remaining spectra. To deepen our insight into the CNN prediction mechanisms, we visualized the patterns recognized by the feature extraction layers of the network as linear plots. Although CNNs are generally referred to as being opaque (which means that we cannot precisely show the specifics of the method), there are ways of depicting how the CNN “sees” the data in subsequent parts of the prediction process. In our study, we extracted the outputs of the classification layers of the CNN, specifically the first, second, and last dense layers, immediately before the final classifier (called Softmax, see Supplementary Figure S1). Interestingly, in the received plot images, no specific patterns are recognizable to the human eye (Supplementary Images S14, S15, and S16). However, the resulting classifications into 5 classes of the samples presented in these figures are 100% correct. This shows that CNN can detect otherwise unidentifiable spectral differences. Moreover, it proves that the CNN-based approach in spectroscopic data classification is an excellent accessory tool in PC diagnostics.

Our finding regarding the DNA methylation profiles in PC translates into the analysis of EV compounds, such as circulating tumor DNA (ctDNA) [14, 60]. EVs are the major carriers of the single-stranded and double-stranded DNA in a liquid biopsy [60] that might be used for the serum-based early diagnostics of PC [14].

Overexpression of β-sheet-rich proteins in PC tumor cells leads directly to the presence of these proteins in circulating tumor-derived EVs. Recent findings suggest that secondary structural signatures, specifically the content of β-sheet secondary structures in vesicle proteins, can be used as diagnostic biomarkers for noninvasive screening for PC [49]. We complement these reports by specifying the characteristics of the β-sheet protein contents to three main PC groups, differentiating the liquid biopsy testing results. Blood tests relying on individual protein markers are characterized by low sensitivity and specificity in cancer diagnostics [61]. The use of VS methodology for the characterization of global proteins derived from cancerous cells and carried by EVs in the bloodstream might be a better candidate for early PC diagnosis. Our findings establish the protein spectroscopic profile of PC subtypes.

The results of VS studies on DNA methylation or β-sheet proteins in PC are important for yet another reason. The lack of sensitive and specific minimal residual disease (MRD) monitoring methods in PC patients who have undergone curative surgery is currently a major problem [62]. It was shown that ctDNA analysis in a liquid biopsy might be a good solution for MRD monitoring using genetic techniques [62]. The idea of using VS methodology in MRD monitoring of PC patients is justified and might be suitable because of its holistic approach.

## Conclusions

It is characteristic of the VS methodology that the results obtained are not specific to single proteins or DNA methylation locations. The information is general. What makes VS successful in recognizing specific malignant entities is the spectral data “fingerprint”, made by combined signals from all the molecules in the studied samples. Combined with CNN technology, VS is a powerful tool for the detection of multiple cancers [58]. The fingerprint of DNA methylation and β-sheet intracellular proteins established by our results varies among AVAC, cPDAC, and IPMC and differentiates these tumors with 94% accuracy.

The major barrier in translating molecular imaging methods based on VS into clinical practice is the extent of knowledge and experience required for successful measurements and analysis. This “know-how” is not easily available. Our study sheds new light on ways of enabling this translation using CNN-based methodology. The development of new diagnostic technologies has thus become a possibility. The use of complementary to RHM vibrational spectroscopic methods such as SERS or not as sophisticated but commonly accessible ATR-FTIR might be the future of serum-based PC early diagnostics [14]. For proper utilization of these VS techniques in serum liquid biopsy testing, an understanding of the molecular structure and composition of PC cells is needed.

## Supplementary Information

Below is the link to the electronic supplementary material.

Supplementary file1 (DOCX 6880 KB)

## Data Availability

All data obtained during the studies are available from the corresponding authors upon reasonable request. The source codes for the CNN training, CNN-based prediction with map plotting, and CNN extracted features plotting are accessible via the Code Ocean platform under https://codeocean.com/capsule/4784100/tree/v2, https://codeocean.com/capsule/8578161/tree/v3, and https://codeocean.com/capsule/0700141/tree/v1, respectively.

## Abbreviations

PC: Pancreatic cancer
RHM: Raman hyperspectral mapping
VS: Vibrational spectroscopy
RS: Raman spectroscopy
NMF: Nonnegative matrix factorization
PCA: Principal component analysis
HCA: Hierarchical cluster analysis
CNN: Convolutional neural network
IPMN: Intraductal papillary mucinous neoplasm
PanIN: Pancreatic intraepithelial neoplasia
cPDAC: Conventional pancreatic ductal adenocarcinoma
AVAC: Adenocarcinoma of the ampulla of Vater
IPMC: Carcinoma derived from IPMN
FTIR: Fourier transform infrared spectroscopy
ATR-FTIR: Attenuated total reflection Fourier transform infrared spectroscopy
SERS: Surface-enhanced Raman spectroscopy
