# Supplementary material for: Variabilities in global DNA methylation and β-sheet richness establish spectroscopic landscapes among subtypes of pancreatic cancer
Source: Eur J Nucl Med Mol Imaging. 2023 Feb 9;50(6):1792–810. doi: 10.1007/s00259-023-06121-7 (PMC10119063; doi:10.1007/s00259-023-06121-7)
Supplement: Supplementary file 1 — Supplementary file1 (DOCX 6880 KB) [file 259_2023_6121_MOESM1_ESM.docx]

**Variabilities in global DNA methylation and β-sheet richness establish spectroscopic landscapes among subtypes of pancreatic cancer**

Krzysztof Szymoński^1,2*^, Ewelina Lipiec^3^, Kamila Sofińska^3^, Katarzyna Skirlińska-Nosek^3^, Michał Czaja^3^, Sara Seweryn^3^, Natalia Wilkosz^3^, Giovanni Birarda^4^, Federica Piccirilli^4^, Lisa Vaccari^4^, Łukasz Chmura^1,2^, Joanna Szpor^1,2^, Dariusz Adamek^1^, Marek Szymoński^3^

^1^ Department of Pathomorphology, Jagiellonian University Medical College, Cracow, Poland

^2^ Department of Pathomorphology, University Hospital, Cracow, Poland

^3^ M. Smoluchowski Institute of Physics, Jagiellonian University, Cracow, Poland

^4^ Elettra-Sincrotrone Trieste S.C.p.A, Trieste, Italy


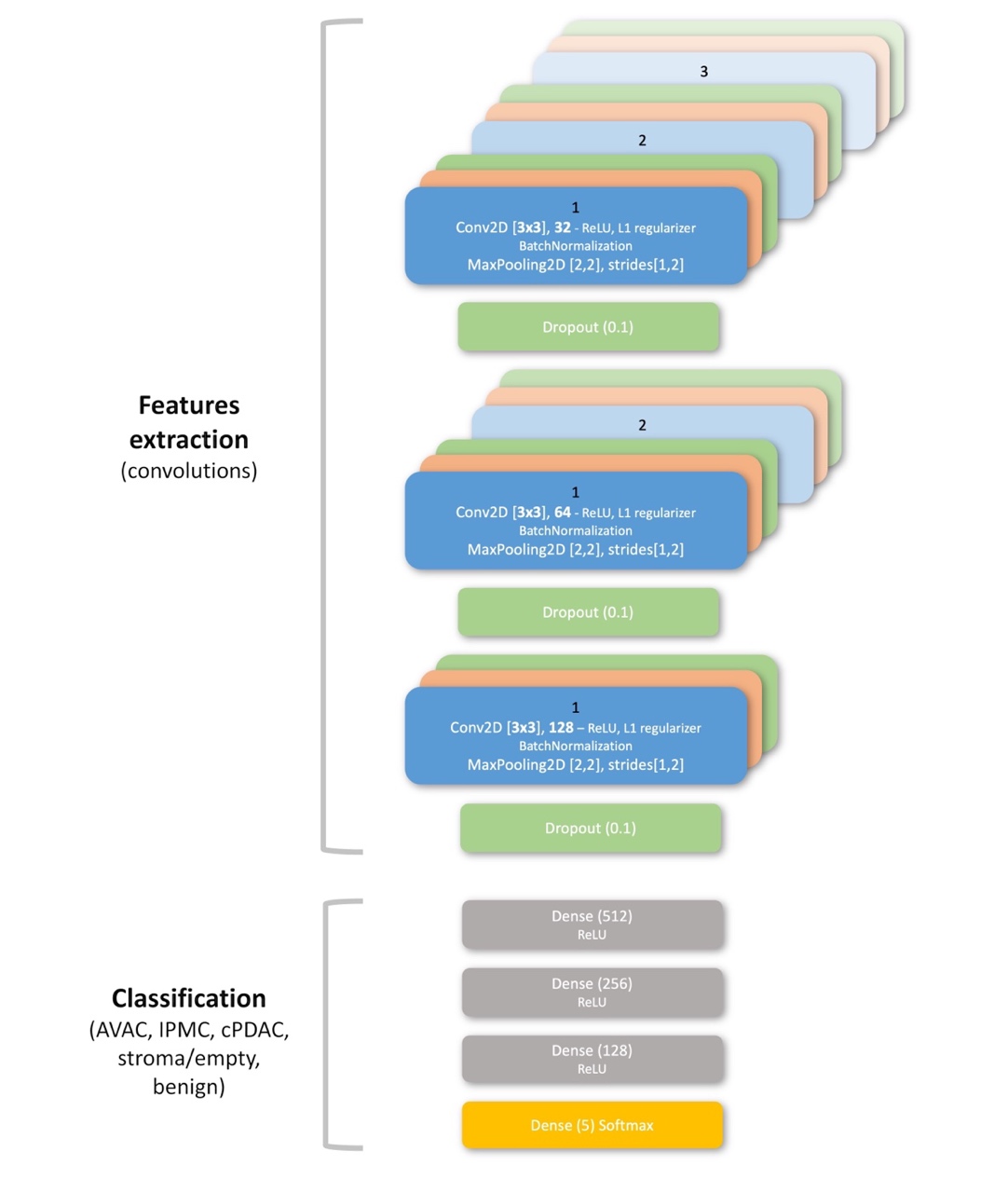


*Supplementary Figure S1. Custom CNN architecture designed for training and testing in differentiating pancreatic tumors of different origins (AVAC, cPDAC, and IPMC).* The CNN was composed of 3 sets of convolutional blocks with 32 filters (kernel size [3x3]), combined with batch normalization, and max pooling (pool size [2,2], stride [1,2]), then a dropout (0.1) layer, followed by 2 sets of similar convolutional blocks but with 64 filters, another dropout (0.1), and a single block with 128 filters, and a dropout (0.1). The L1 kernel regularizer and a ReLu activation were used for each convolutional layer. The CNN model was finalized with fully connected classification layers.

*
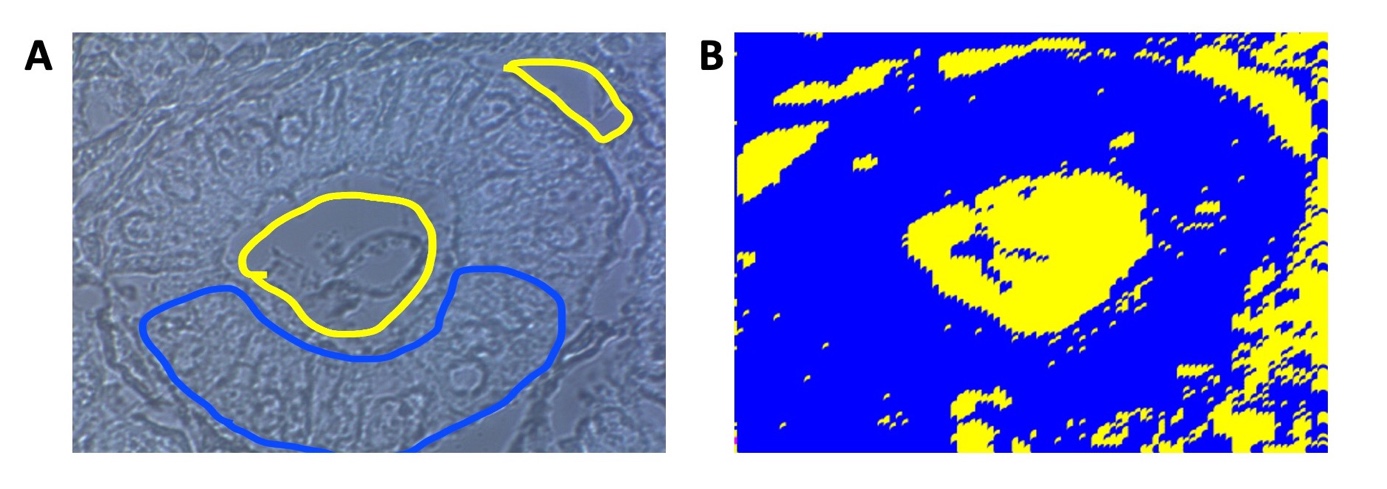
*

*Supplementary Figure S2. The annotation process of CNN training dataset.* On unstained tissue slides, a pathologist marked areas of cancer cells and the tumor stroma (or clean space) with different labels. The spectra from corresponding areas on the RHM map were extracted as the training dataset. As depicted in **(A)** only a fraction of the total areas was marked for each label (approximately 25% of spectra was extracted for the CNN training dataset), leaving the rest for the CNN as a validation dataset. The CNN model generalized well on the whole RHM map **(B)**, as confirmed by the pathologist.


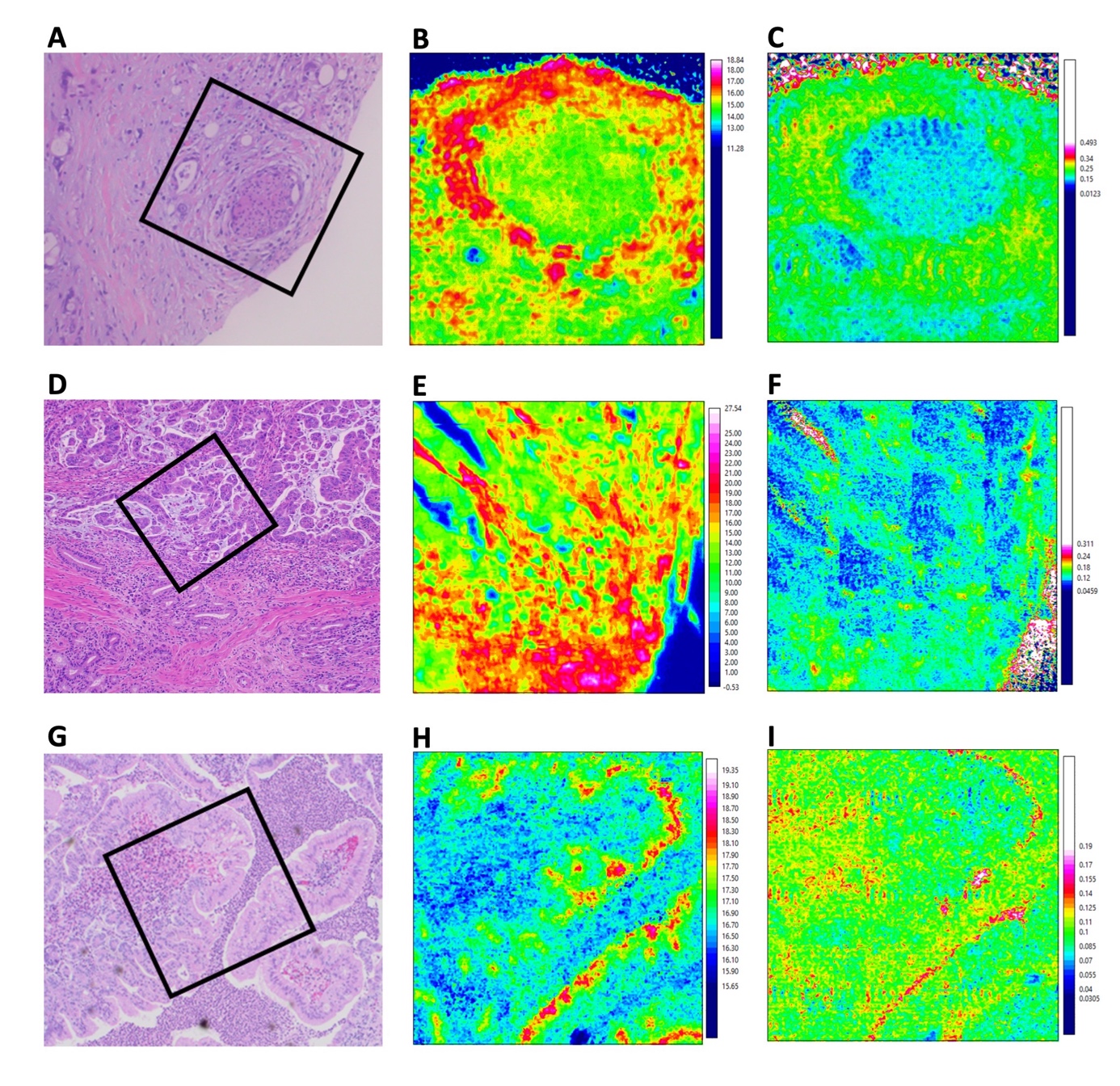


Supplementary Figure S3. Distribution of characteristic chemical substances, such as proteins and nucleic acids, in PC tissue samples. **(A, D, G)** Optical images of PC tissues (cPDAC, AVAC, and IPMC, respectively) stained with hematoxylin-eosin and marked areas of infrared maps acquisition (H&E stain, original magnification x100). **(B, E, H)** The distribution of **protein concentration** is presented by false-color maps of amide I and amide II bands intensity (1700-1500 cm^-1^). **(C, F, I)** The distribution of **nucleic acid concentration** by false-color maps of ν_as_(PO_2_^-^) (1250-1220 cm^-1^) is depicted.


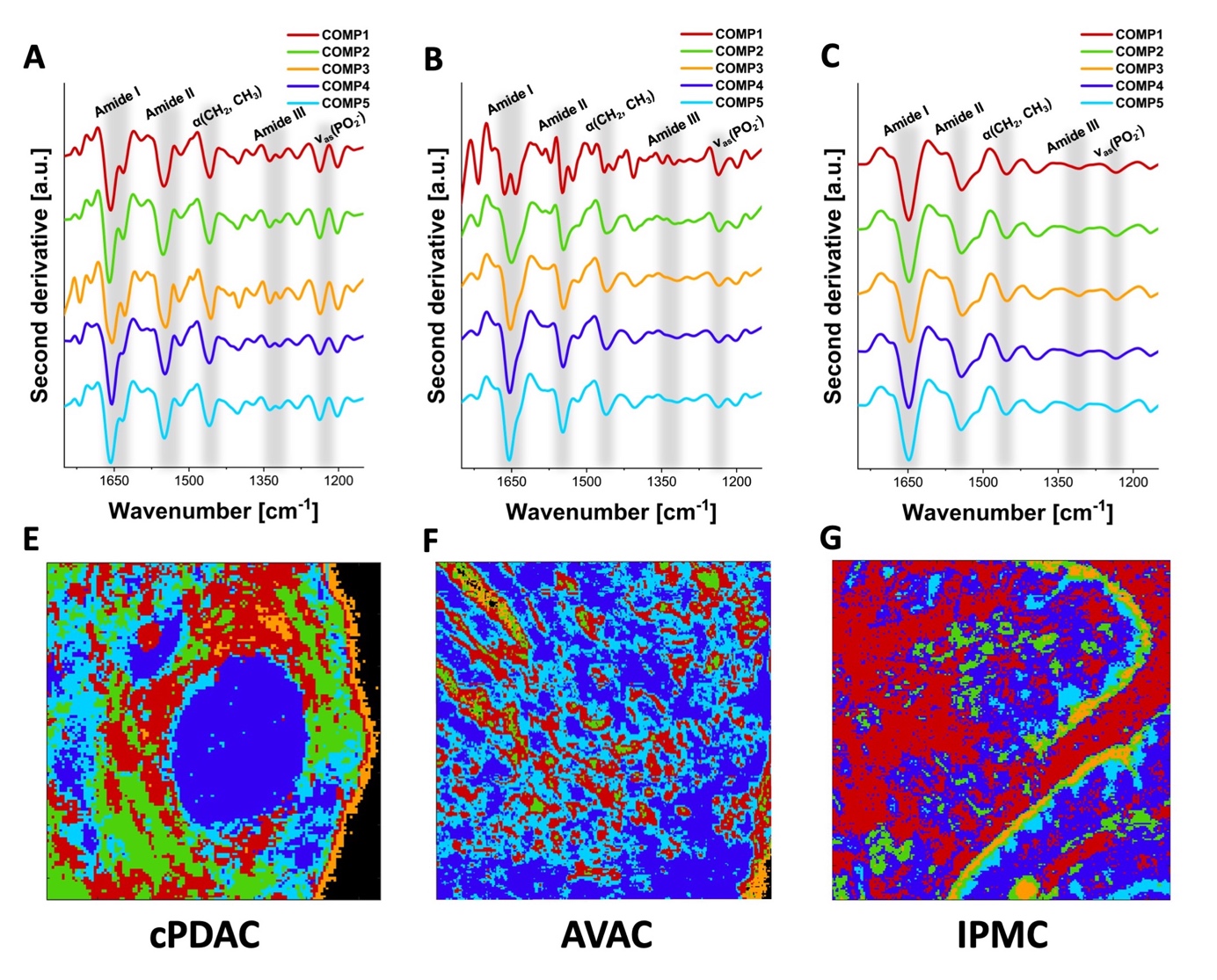


Supplementary Figure S4*. The distribution of proteins and nucleic acids measured in PC tumors.* **(A, B, C)** Second derivatives of mean FTIR spectra that correspond to particular HCA components, with highlighted characteristic bands of proteins and nucleic acids. **(E, F, G)** FTIR false-color maps depicting the HCA components, with corresponding pixel colors.


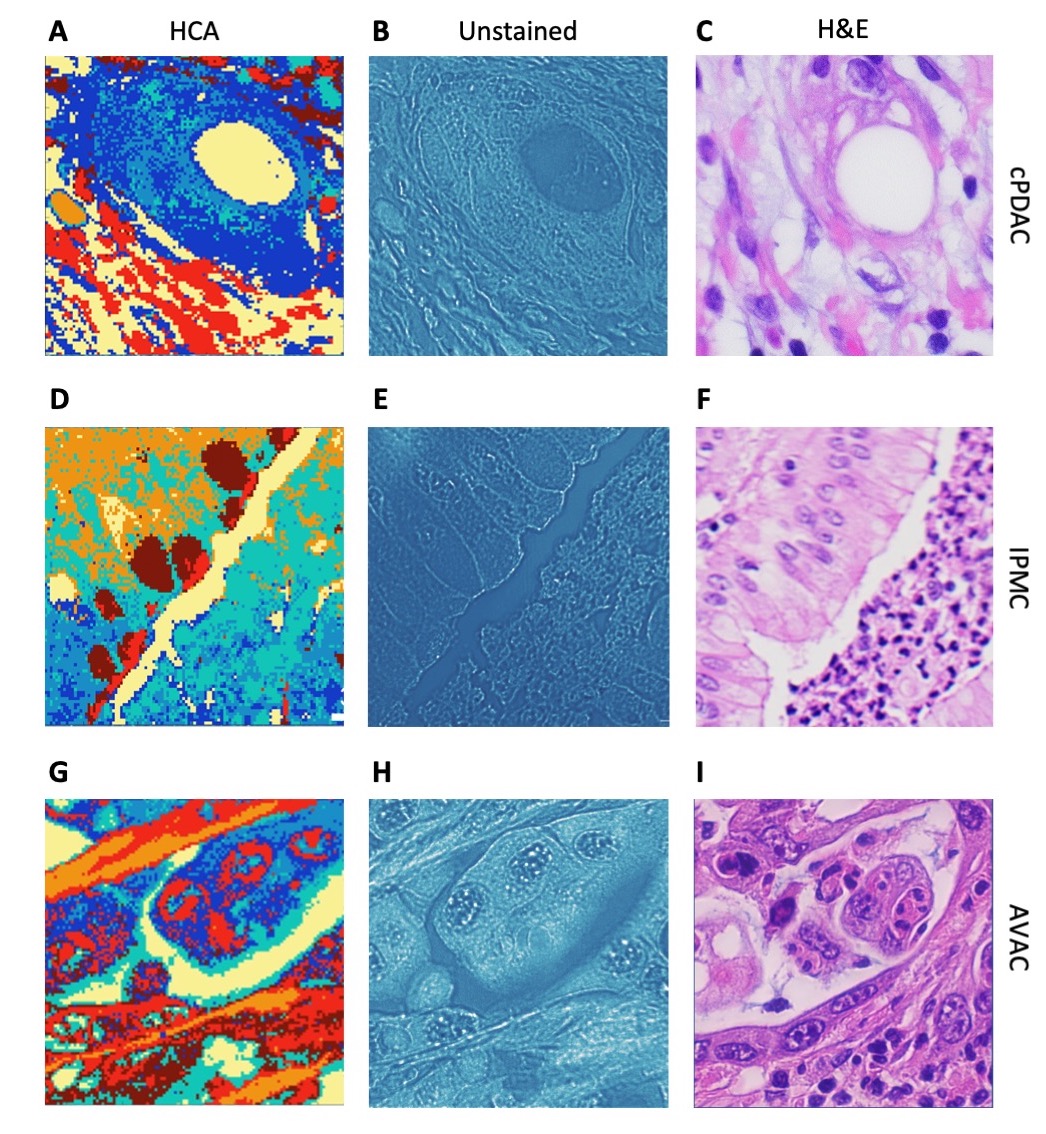


*Supplementary Figure S5. Comparison of HCA maps and corresponding unstained, and H&E stained tissue slides.* **(A, B, C)** cPDAC, **(D, E, F)** IPMC, and **(G, H, I)** AVAC. The H&E sections (C, F, I) were sliced from the FFPE tissue blocks before the unstained sections (B, E, H) (more superficial), thus the corresponding tissue elements might slightly differ from the latter. The RHM maps were collected from unstained sections, and consequently, these are morphologically similar to the HCA elements. The individual cancer cells, with cellular nuclei and the cytoplasm, are seen. (original magnification x600)


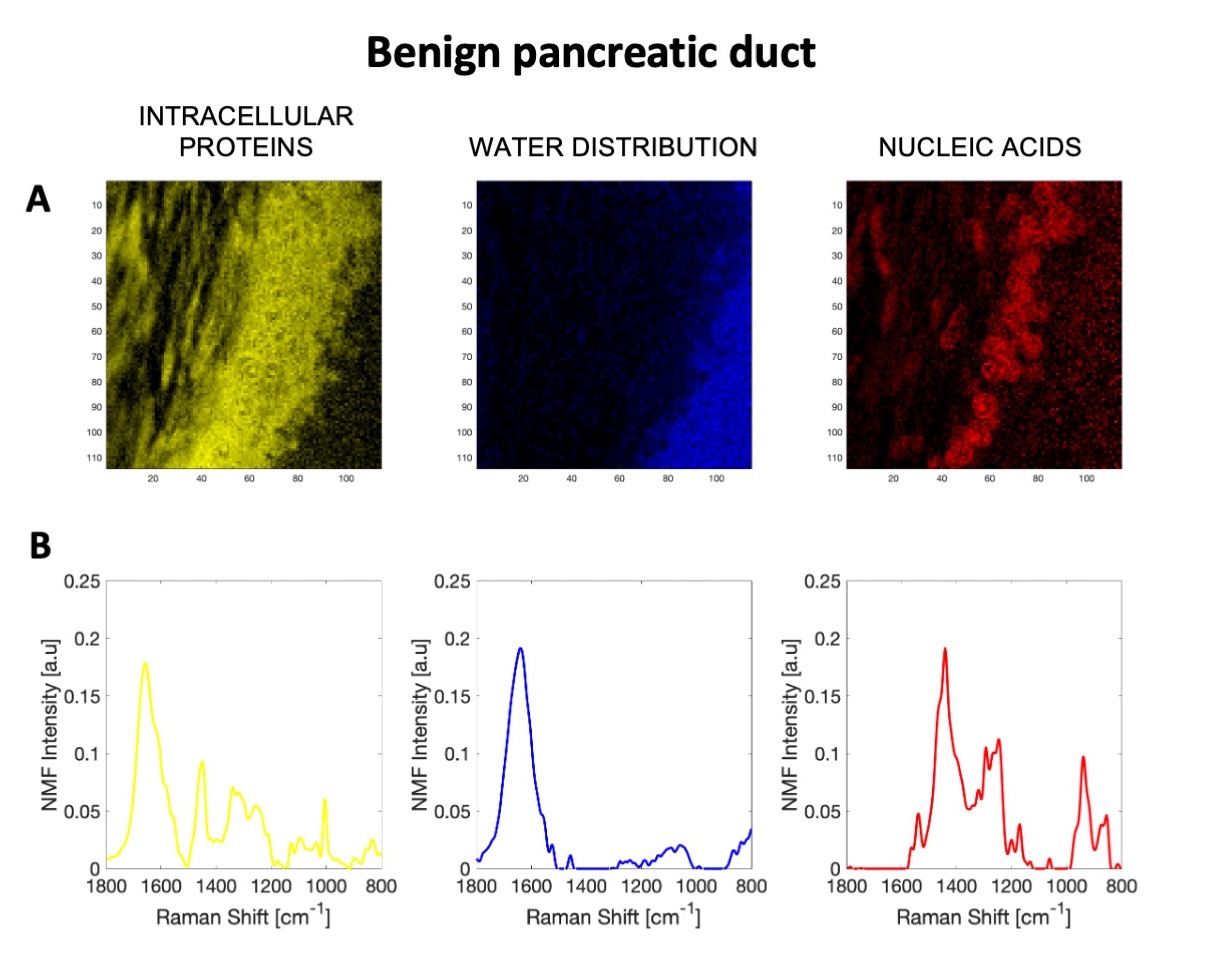


Supplementary Figure S6*. The NMF maps and NMF components of the benign pancreatic duct tissue sample.* **(A)** distribution of the NMF components, highlighting proteins, water, and nucleic acids, and **(B)** corresponding plots of these NMF components.

**Amid I**


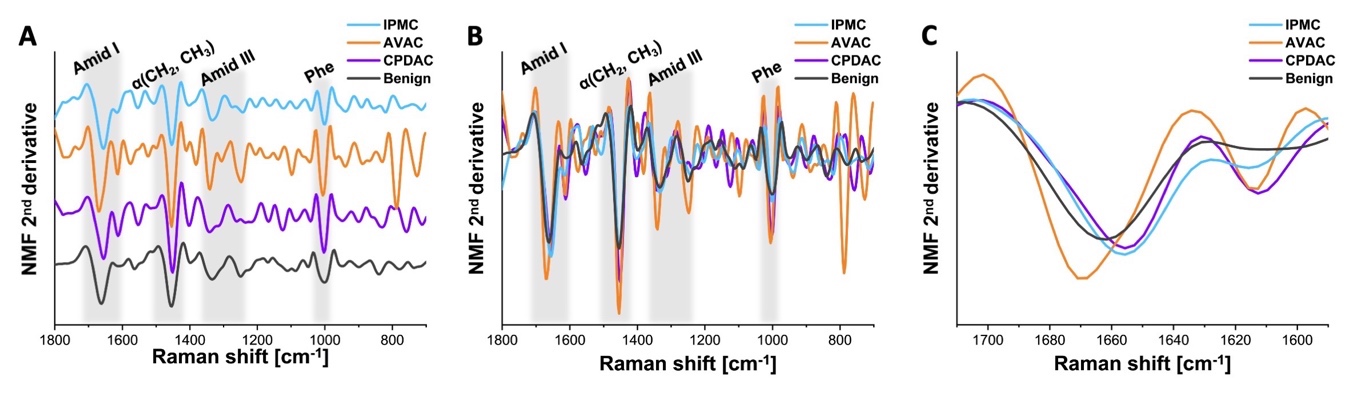


*Supplementary Figure S7. 2^nd^ derivatives of NMF components characteristic for PC tumors’ proteins*  The bands related to vibrations specific to proteins are highlighted in gray. The alterations of protein patterns in studied PC tumors reveal unique molecular phenotypes for each PC type. To demonstrate spectral differences 2^nd^ derivatives are plotted in a stack **(A)** and overlapping **(B)**, additionally, the zoomed area of the Amide I spectral region **(C)** is presented.


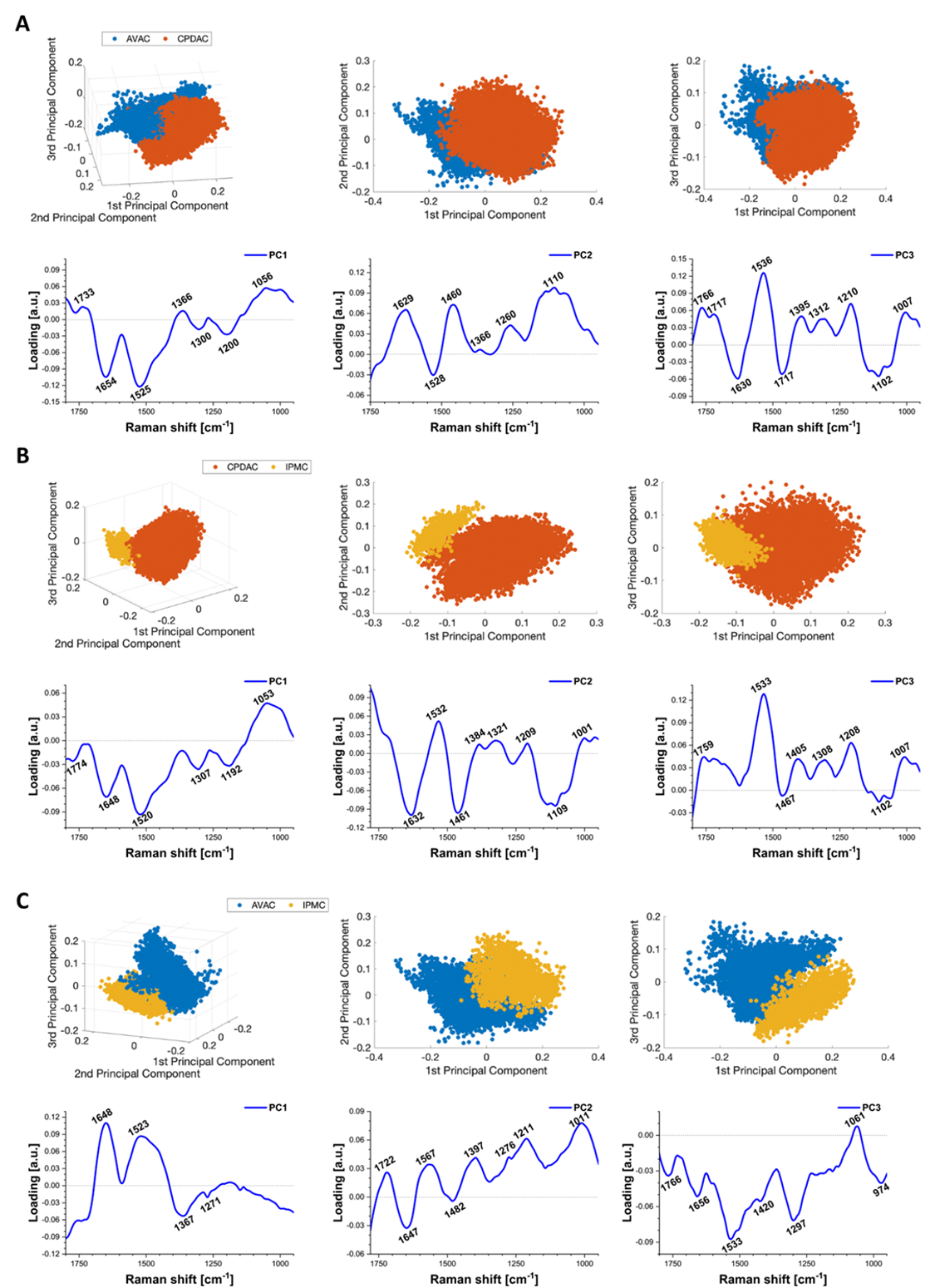


*Supplementary Figure S8. PCA 3D and 2D scores plots of spectra extracted from cancerous cells’ nuclei of all the investigated tissue sections. A pairwise comparison is presented with the corresponding loadings plots, specifically, the data from cPDAC is compared with AVAC* ***(A)****, cPDAC with IPMC* ***(B)****, and AVAC with IPMC samples* ***(C)****.*


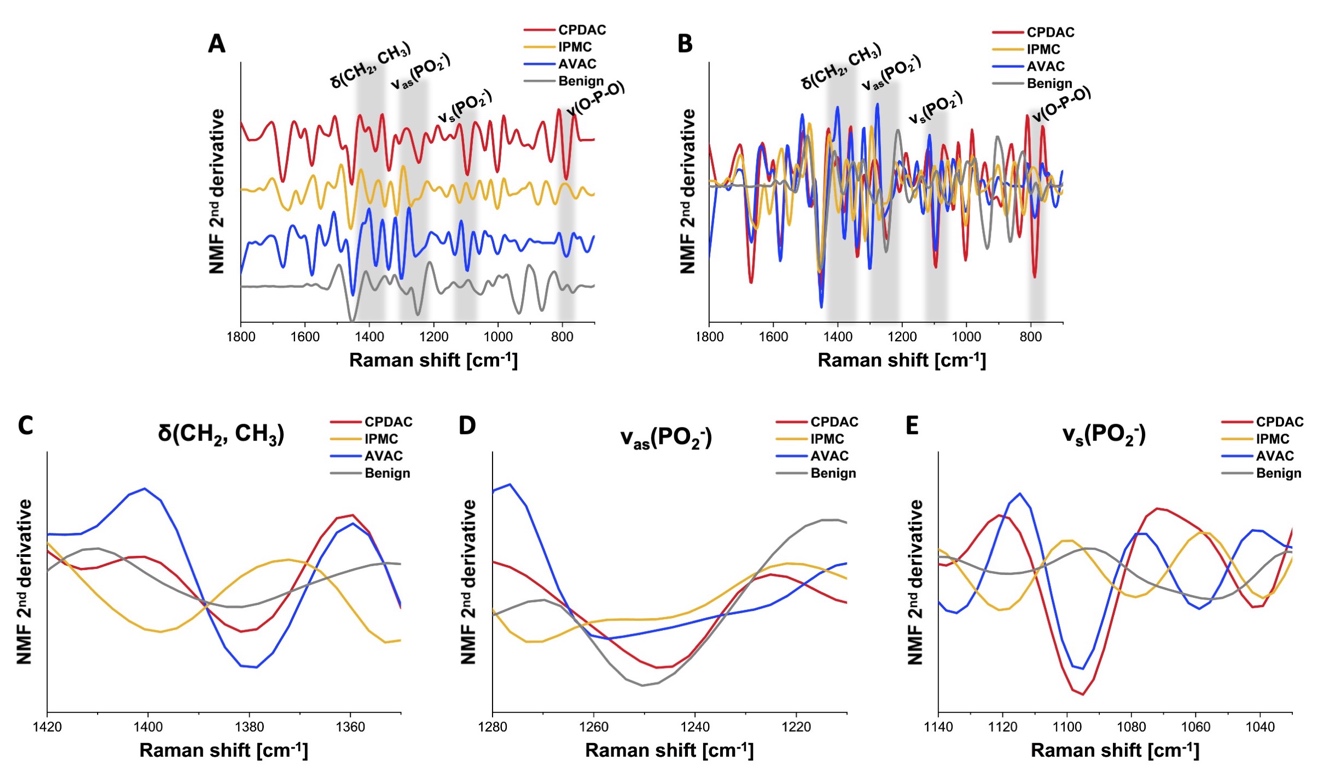


*Supplementary Figure S9. 2^nd^ derivatives of NMF components characteristic for PC tumors’ DNA conformation and methylation.* The bands related to vibrations specific to DNA are highlighted in gray. The alterations of DNA patterns in studied PC tumors reveal unique molecular phenotypes for each PC type. To demonstrate spectral differences 2^nd^ derivatives are plotted in a stack **(A)** and overlapping **(B)**, additionally, zoomed spectral ranges of methyl and methylene **(C)**, *phosphate asymmetric* ***(D),*** *and phosphate symmetric* ***(E)*** *stretching are presented.*


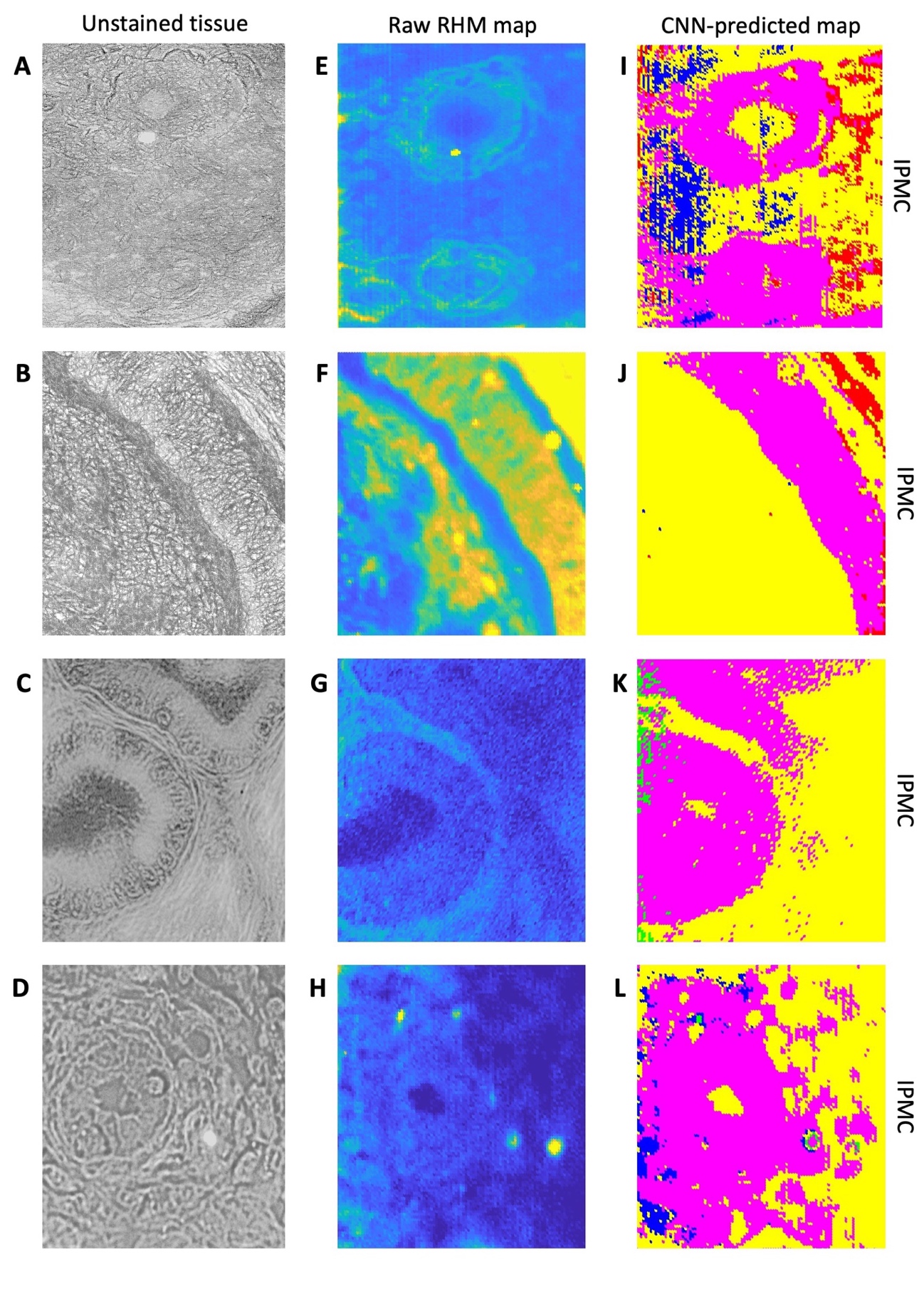


*Supplementary Figure S10. CNN-predicted maps of IPMC.* **(A, B, C, D)** Unstained, optical microscopy slide images of IPMC tumors (unstained tissues, original magnification x600, grayscale, contrast-enhanced). **(E, F, G, H)** Raw, unprocessed RHM maps of IPMC. **(I, J, K, L)** Prediction map images as generated by the CNN, which classified each PC tumor of different origin with pixel colors, specifically the red color for cPDAC, blue for AVAC, and magenta for IPMC. The yellow color was used to mark stroma/empty class, and the green color for benign pancreatic tissue. CNN handled the distinction of the origin of PC tumors efficiently and accurately.

~~
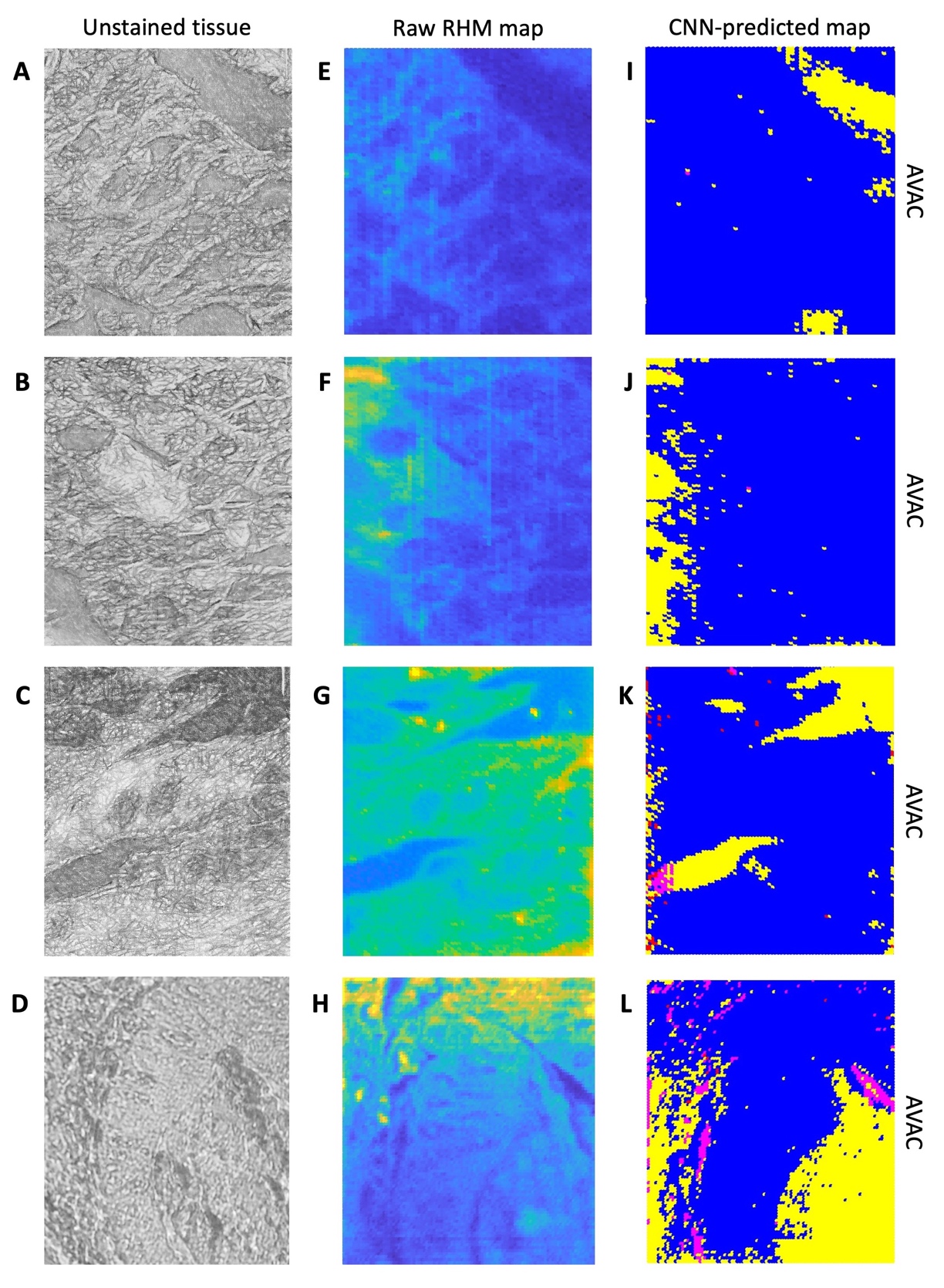
~~

*Supplementary Figure S11. CNN-predicted maps of AVAC.* **(A, B, C, D)** Unstained, optical microscopy slide images of AVAC tumors (unstained tissues, original magnification x600, grayscale, contrast-enhanced). **(E, F, G, H)** Raw, unprocessed RHM maps of AVAC. **(I, J, K, L)** Prediction map images as generated by the CNN, which classified each PC tumor of different origin with pixel colors, specifically the red color for cPDAC, blue for AVAC, and magenta for IPMC. The yellow color was used to mark stroma/empty class, and the green color (not shown in the figures) for benign pancreatic tissue. CNN handled the distinction of the origin of PC tumors efficiently and accurately.


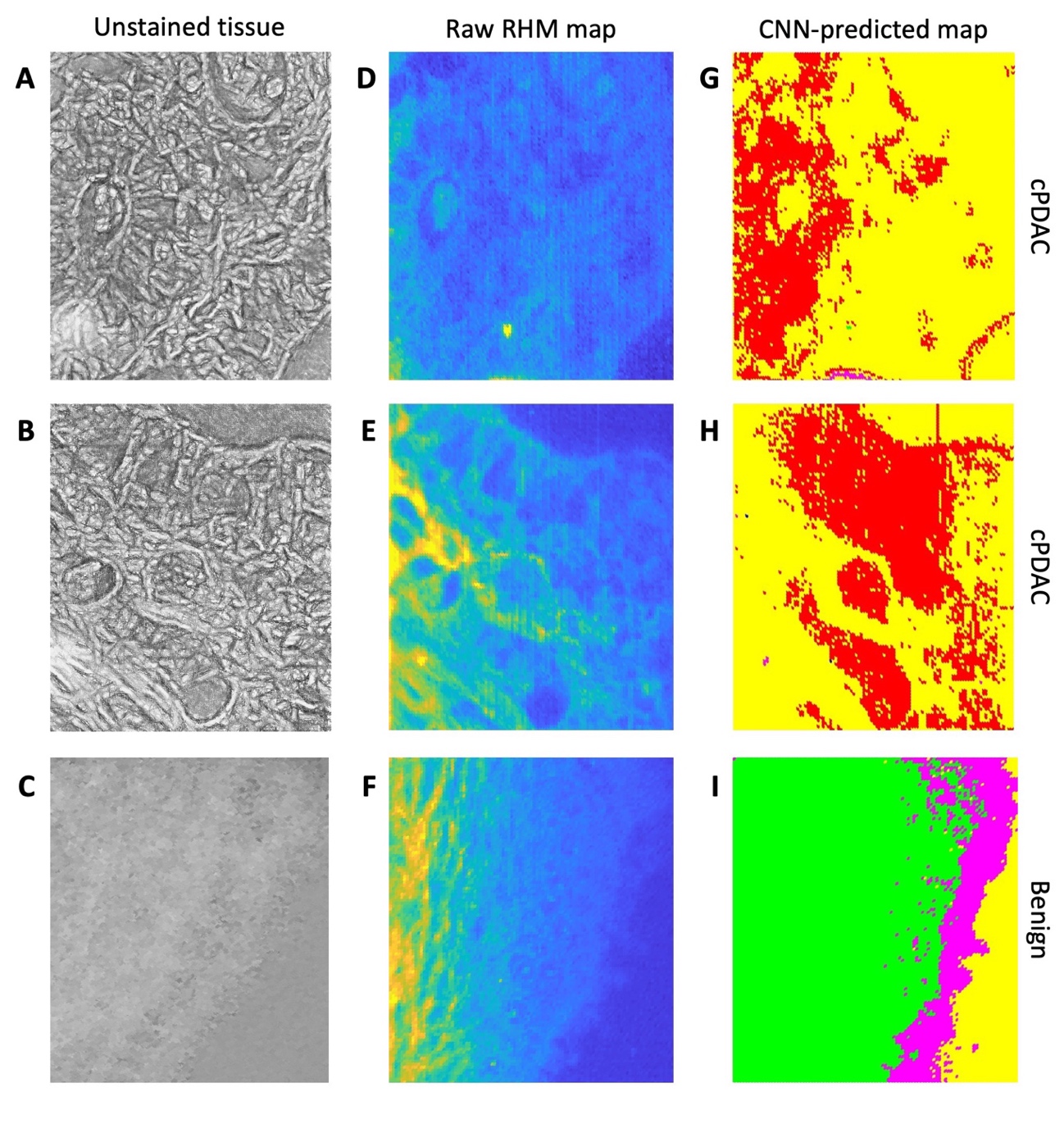


*Supplementary Figure S12. CNN-predicted maps of cPDAC and benign pancreatic tissue.* **(A, B)** Unstained, optical microscopy slide images of cPDAC tumors and **(C)** benign pancreatic tissue (unstained tissues, original magnification x600, grayscale, contrast-enhanced). **(D, E)** Raw, unprocessed RHM maps of cPDAC and **(F)** benign pancreatic tissue. **(G, H, I)** Prediction map images as generated by the CNN, which classified each PC tumor of different origin with pixel colors, specifically the red color for cPDAC, blue for AVAC (not shown in the figures), and magenta for IPMC. The yellow color was used to mark stroma/empty class, and the green color for benign pancreatic tissue. CNN handled the distinction of the origin of PC tumors efficiently and accurately.

**
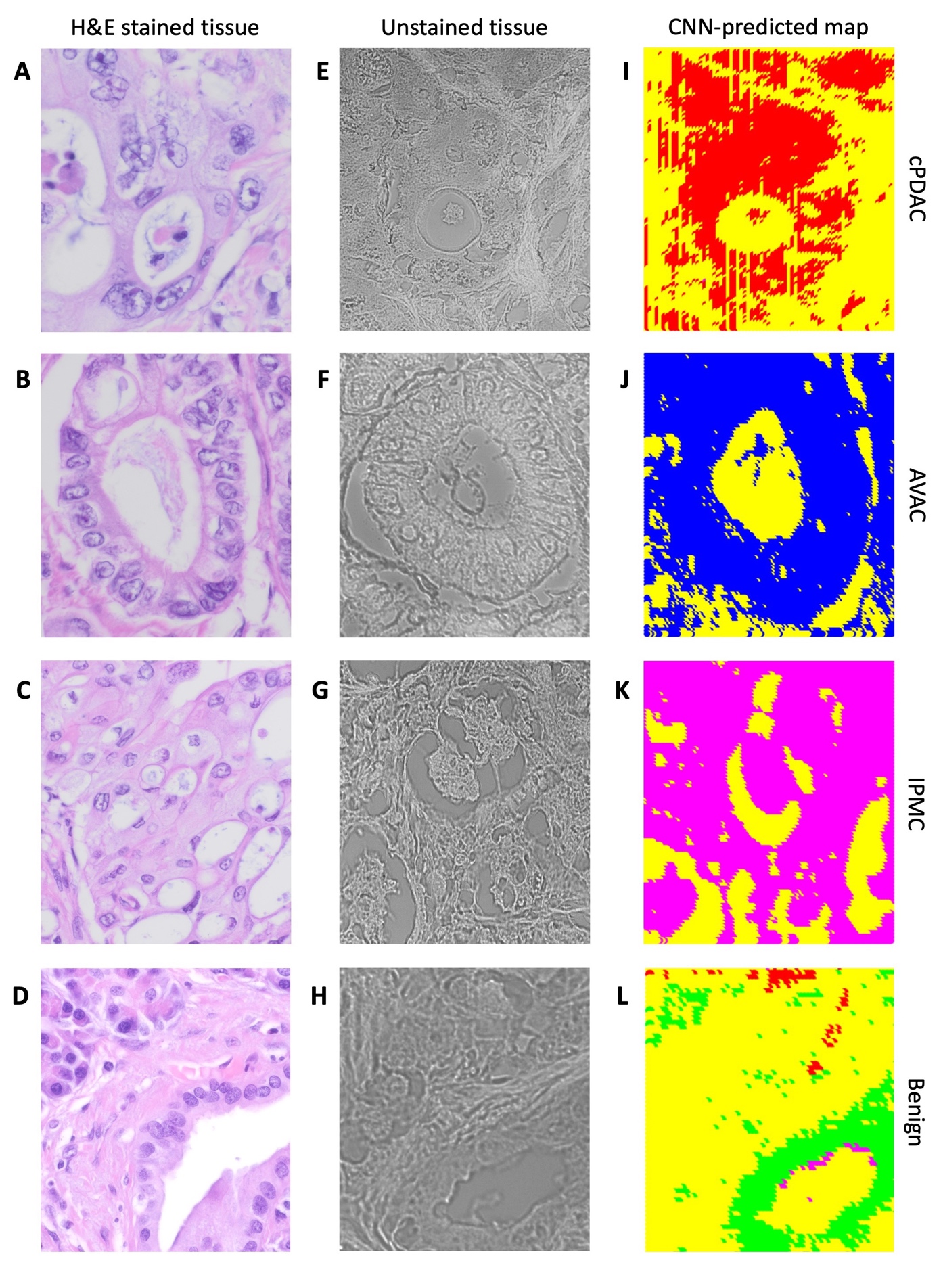
**

*Supplementary Figure S13. CNN-predicted maps of new tissues from cPDAC, AVAC, IPMC, and benign pancreatic duct, validated by the CNN after its training completion.* Corresponding H&E-stained and unstained tissue images show remarkable similarities in cancer cells and glandular structures with the CNN-generated map images. Notice that H&E slides might be slightly different, as the tissues for H&E staining were sliced from FFPE blocks before the tissues for RHM measurements (unstained and CNN).


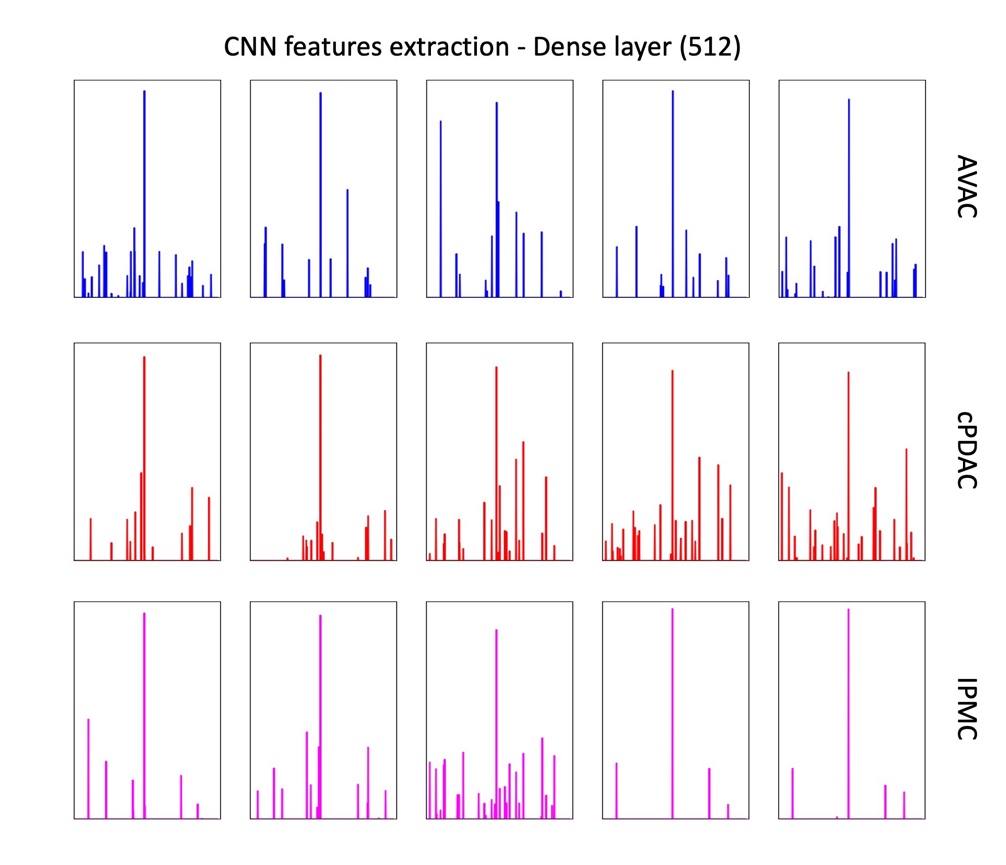


*Supplementary Figure S14. Spectral features extracted by the CNN's dense layer with 512 neurons.* Plots present values from illustrative spectra of each PC tumor type as seen by the CNN at the beginning of the classification process.


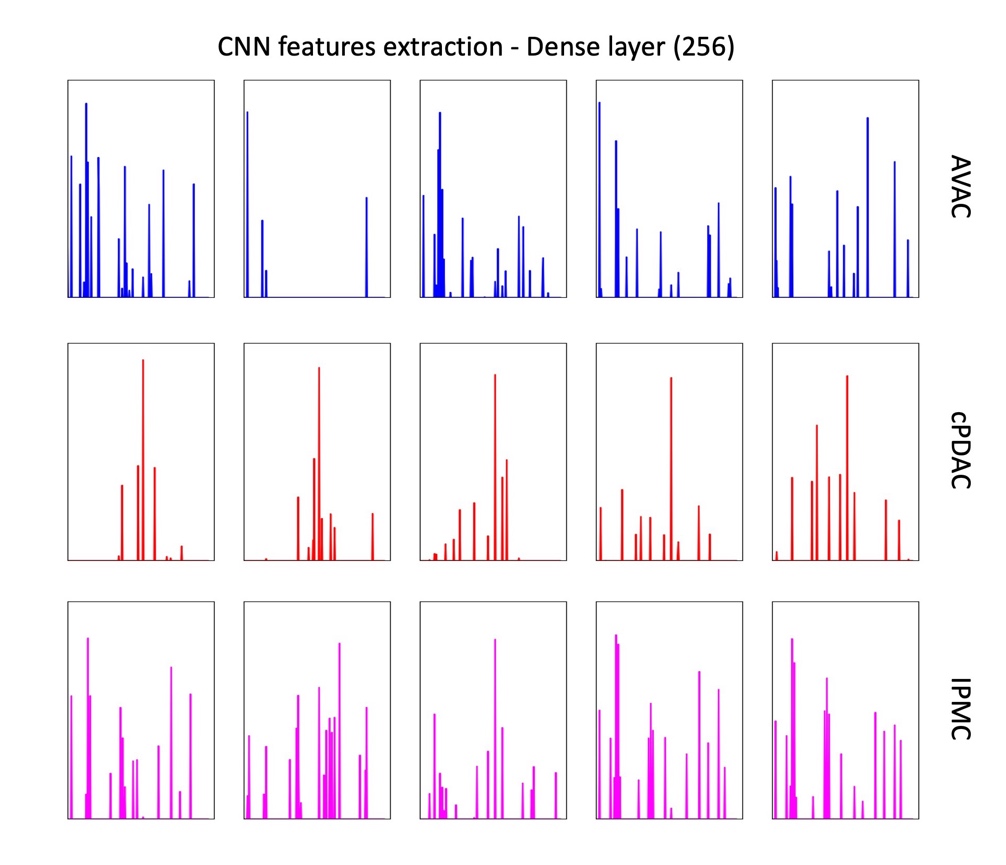


*Supplementary Figure S15. Spectral features extracted by the CNN's dense layer with 256 neurons.* Plots present values from illustrative spectra of each PC tumor type as seen by the CNN at halfway through the classification process.


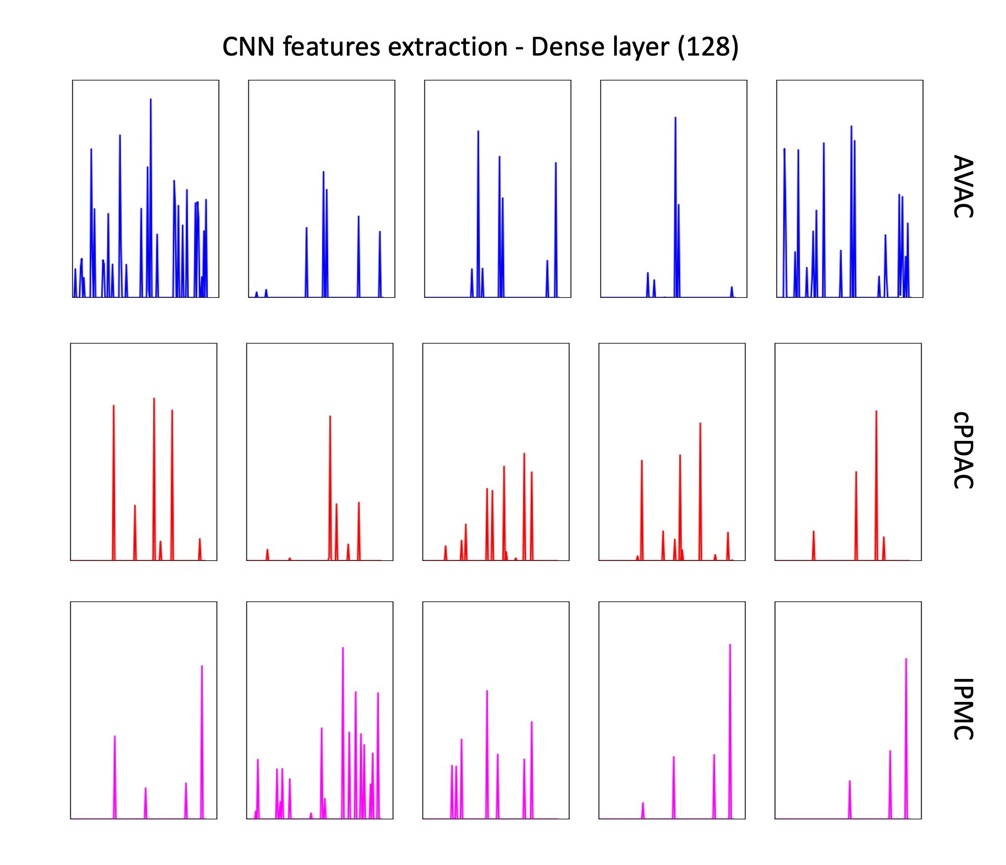


*Supplementary Figure S16. Spectral features extracted by the CNN's dense layer with 128 neurons.* Plots present values from illustrative spectra of each PC tumor type as seen by the CNN at the end of the classification process, just before the Softmax classifier into 5 classes.

*Supplementary Table S1. Characteristics of patients included in the study.* IPTN, Intraampullary papillary tubular neoplasm; IPMN, Intraductal papillary mucinous neoplasm; AJCC, American Joint Committee on Cancer staging classification 8^th^ edition;

| **#** | **Gender** | **Age** | **Tumor location** | **Diagnosis by WHO 5^th^ edition** | **Grade** | **Stage AJCC 8^th^** |
| --- | --- | --- | --- | --- | --- | --- |
| 1 | M | 53 | Ampulla of Vater | IPTN with an associated carcinoma  intestinal type | G2 | pT2 |
| 2 | F | 83 | Ampulla of Vater | Ampullary ductal carcinoma  pancreatobiliary type | G2 | pT3b |
| 3 | M | 67 | Common bile duct | Adenocarcinoma of common bile duct  pancreatobiliary type | G2 | pT3 |
| 4 | F | 73 | Ampulla of Vater | IPTN with an associated carcinoma  pancreatobiliary type | G2 | pT1b |
| 5 | F | 63 | Pancreatic head | Ductal adenocarcinoma, NOS | G3 | pT3 |
| 6 | F | 61 | Pancreatic head | Ductal adenocarcinoma, NOS | G2 | pT2 |
| 7 | M | 67 | Pancreatic head | Ductal adenocarcinoma, NOS | G2 | pT2 |
| 8 | M | 51 | Pancratic tail | Ductal adenocarcinoma, NOS | G2 | pT2 |
| 9 | M | 61 | Pancreatic head | Ductal adenocarcinoma, NOS | G3 | pT3 |
| 10 | M | 72 | Pancratic tail | IPMN with an associated invasive carcinoma | G2 | pT2 |
| 11 | M | 58 | Pancreatic head | IPMN with an associated invasive carcinoma | G1 | pT1a |
| 12 | M | 76 | Pancreatic head | IPMN with an associated invasive carcinoma | G2 | pT3 |
| 13 | F | 78 | Pancreatic head | IPMN with an associated invasive carcinoma | G3 | pT2 |
| 14 | F | 68 | Pancreatic head | IPMN with an associated invasive carcinoma | G2 | pT2 |
| 15 | F | 54 | Ampulla of Vater | Ampullary ductal carcinoma  pancreatobiliary type | G3 | pT2 |
| 16 | M | 67 | Pancreatic head | IPMN with an associated invasive carcinoma | G3 | pT2 |
| 17 | M | 63 | Pancreatic head | Ductal adenocarcinoma, NOS | G3 | pT2 |

*Supplementary Table S2. Raman bands assignment of PC tissue spectral features.*

| **Raman shift / Wavenumber**  **[cm^-1^]** | **Functional groups  vibration** | **Biomolecule assignment** | **Reference** |
| --- | --- | --- | --- |
| 1670 | Amide I:  ν(C=O) | Proteins  (antiparallel β-sheet) | (1–3) |
| 1650 | Amide I:  ν(C=O) | Proteins  (a-helix, unstructured coils;  collagen, elastin) | (1–3) |
| 1630 | Amide I:  ν(C=O) | Proteins  (parallel β-sheet) | (1–3) |
| 1551 | Amide II:  ν(C-N), δ(N-H) | Proteins | (4) |
| 1447 | α(CH_2_, CH_3_) | Proteins | (5–8) |
| 1370-1340 | Amide III:  ν(C-N), δ(N-H);  ω(CH_2_,CH_3_) | Nucleic acids,  Proteins (collagen) | (4,6,9,10) |
| 1280 | Amide III | Proteins (α-helix) | (11) |
| 1260-1220 | ν_as_(PO_2_^−^) | DNA | (12) |
| 1240 | Amide III:  ω(CH_2_) | Proteins (collagen) | (10,13) |
| 1100-1050 | ν_s_(PO_2_^−^) | DNA | (9) |
| 1080 | ν(C-N) | Proteins | (9,14,15) |
| 1004 | Symmetric ring breathing | Proteins (Phe) | (6,7,9) |
| 850 | Ring breathing  ν(C-C) | Proteins (Tyrosine)  Proteins (collagen) | (6,9,10) |
| 832 | Out-of-plane ring breathing  ν(O-P-O) | Proteins (Tyrosine)  DNA | (10) |
| 814 | ν(O-P-O) | DNA | (9,16) |
| 760 | ν_s_(O-P-O),  Symmetric ring breathing | DNA  Proteins (Tryptophan) | (6,9,10) |
| ν – stretching (s – symmetrical; as – asymmetrical); α – scissoring; ω – wagging; δ – bending | | | |

**References**

1. Malini R, Venkatakrishna K, Kurien J, Pai KM, Rao L, Kartha VB, et al. Discrimination of normal, inflammatory, premalignant, and malignant oral tissue: a Raman spectroscopy study. Biopolymers. 2006;81:179–93.

2. Caspers PJ, Lucassen GW, Carter EA, Bruining HA, Puppels GJ. In vivo confocal Raman microspectroscopy of the skin: noninvasive determination of molecular concentration profiles. J Invest Dermatol. 2001;116:434–42.

3. Rygula A, Majzner K, Marzec KM, Kaczor A, Pilarczyk M, Baranska M. Raman spectroscopy of proteins: a review. Journal of Raman Spectroscopy. 2013;44:1061–76.

4. Zhu F, Isaacs NW, Hecht L, Barron LD. Raman optical activity: a tool for protein structure analysis. Structure. 2005;13:1409–19.

5. Crow P, Barrass B, Kendall C, Hart-Prieto M, Wright M, Persad R, et al. The use of Raman spectroscopy to differentiate between different prostatic adenocarcinoma cell lines. Br J Cancer. 2005;92:2166–70.

6. Buschman HP, Deinum G, Motz JT, Fitzmaurice M, Kramer JR, van der Laarse A, et al. Raman microspectroscopy of human coronary atherosclerosis: Biochemical assessment of cellular and extracellular morphologic structures in situ. Cardiovascular Pathology. 2001;10:69–82.

7. Krafft C, Sobottka SB, Schackert G, Salzer R. Near infrared Raman spectroscopic mapping of native brain tissue and intracranial tumors. Analyst. 2005;130:1070–7.

8. Penel G, Delfosse C, Descamps M, Leroy G. Composition of bone and apatitic biomaterials as revealed by intravital Raman microspectroscopy. Bone. 2005;36:893–901.

9. Notingher I, Verrier S, Haque S, Polak JM, Hench LL. Spectroscopic study of human lung epithelial cells (A549) in culture: living cells versus dead cells. Biopolymers. 2003;72:230–40.

10. Stone N, Kendall C, Smith J, Crow P, Barr H. Raman spectroscopy for identification of epithelial cancers. Faraday Discussions. 2004;126:141.

11. Verrier S, Notingher I, Polak JM, Hench LL. In situ monitoring of cell death using Raman microspectroscopy. Biopolymers. 2004;74:157–62.

12. Malek K, Wood BR, Bambery KR. FTIR Imaging of Tissues: Techniques and Methods of Analysis. 2014. page 419–73.

13. Dukor RK. Vibrational Spectroscopy in the Detection of Cancer. Handbook of Vibrational Spectroscopy. John Wiley & Sons, Ltd; 2006.

14. Huang Z, McWilliams A, Lam S, English J, McLean DI, Lui H, et al. Effect of formalin fixation on the near-infrared Raman spectroscopy of normal and cancerous human bronchial tissues. Int J Oncol. 2003;23:649–55.

15. Stone N, Kendall C, Shepherd N, Crow P, Barr H. Near-infrared Raman spectroscopy for the classification of epithelial pre-cancers and cancers. Journal of Raman Spectroscopy. 2002;33:564–73.

16. Borchman D, Tang D, Yappert MC. Lipid composition, membrane structure relationships in lens and muscle sarcoplasmic reticulum membranes. Biospectroscopy. 1999;5:151–67.
